# The Impact of a National Stewardship Policy on the Usage Patterns of Key Monitoring Drugs in a Tertiary Teaching Hospital: An Interrupted Time Series Analysis

**DOI:** 10.3389/fphar.2022.847353

**Published:** 2022-02-18

**Authors:** Haiyan Li, David J. McIver, Wenjing Ji, Jiaxi Du, Hang Zhao, Xiaoni Jia, Yuyao Zhai, Xiaorong Xue

**Affiliations:** ^1^ Department of Pharmacy, Xi’an People’s Hospital (Xi’an Fourth Hospital), The Affiliated Hospital of Northwestern Polytechnical University, Xi’an, China; ^2^ Institute for Global Health Sciences, University of California, San Francisco, San Francisco, CA, United States; ^3^ The Department of Pharmacy Administration and Clinical Pharmacy, School of Pharmacy, Center for Drug Safety and Policy Research, Xi’an Jiaotong University, Xi’an, China; ^4^ Department of Science and Education, Xi’an Mental Health Center, Xi’an, China; ^5^ Department of Pharmacy, Xi’an Mental Health Center, Xi’an, China

**Keywords:** National Key Monitoring Drugs, national stewardship policy, clinical pharmacists, interrupted time series, hospital

## Abstract

**Background:** The management of Key Monitoring Drugs has become one of important aspects to control the growth of pharmaceutical expenditures in China. The first batch of the China National Key Monitoring Drugs (NKMDs) policy was released in July 2019. However, little is known about the impact of the national stewardship on the trends of NKMDs prescribing practice in hospitals, especially in the Northwestern China.

**Methods:** We collected 8-years of monthly NKMDs usage data from a tertiary hospital between 2014 and 2021. A segmented regression model of interrupted time series (ITS) analysis was used to evaluate the Defined Daily Doses (DDDs) and spending trends of ten NMKDs in the hospital throughout the study period. The pre-implementation period was from January 2014 to November 2019 and the post-implementation period was from December 2019 to June 2021.

**Results:** Prior to the implementation of the NKMDs policy, there was an increasing trend both in DDDs and spending for 8 of 10 NKMDs. The interventions managed by clinical pharmacists after the implementation of the national stewardship policy led to a significant decreasing trend of DDDs in the 19 months following implementation, of 430 fewer DDDs per month in total, compared to the pre-implementation period (*p* < 0.001). A similar decrease in spending was seen in the post-implementation period, with a trend of $4,682 less total spending on medications in those months compared to the pre-implementation trend (*p* = 0.003). There was a significant decrease in both monthly DDDs and spending for 6 of the 10 medications in the post-implementation period, while there was a significant increased trend both in monthly DDDs and spending on 1 medication in that period.

**Conclusion:** Using ITS analysis, the total DDDs and spending on 10 NKMDs in this hospital indicated sustained reductions over 19 months after multidimensional interventions under the implementation of the national policy guidance. The national stewardship policy could therefore be considered an effective strategy. Additional comprehensive policies should be introduced to further improve the rational use of NKMDs.

## Introduction

Adjuvant Drugs are defined as agents that aid or increase the action of the principal drug, or that impacts the absorption, mechanism of action, metabolism, or excretion of the primary drug in such a way as to enhance its effects (U.S. National Library of Medicine, 1975; Yang J. et al., 2021a). Adjuvant Drugs do not normally play a major role in terms of therapeutic value or economic cost, but unnecessary prescribing of such medications in the hospitals have become a peculiar phenomenon in China (Han et al., 2016). A survey by Han Shuang showed that unreasonable or off-label use of Adjuvant Drugs occurred in 98% of medical institutions in China (Han et al., 2016). The management of Adjuvant Drugs has become an important aspect of the management of rational drug use in China (Yang J. et al., 2021a). Key Monitoring Drugs are a subset of Adjuvant Drugs, which usually have high prices, consumption rates, and unconfirmed therapeutic effects in clinical application. Key Monitoring Drugs cover a broad range of medications, including drugs that enhance tissue metabolism; vitamins; electrolytes; drugs for enteral and parenteral nutrition; neurotrophic drugs; free radical scavenging drugs; traditional Chinese medicines for promoting blood circulation and removing stasis; and drugs for the auxiliary treatment of liver disease, tumors, and other conditions (Yang J. et al., 2021a). The use of Key Monitoring Drugs has grown rapidly in recent years, owing to their wide applications and the commercial promotion of these drugs (Yang J. et al., 2021a). Key Monitoring Drugs have become a major part of clinical drug consumption but are essentially unavailable outside China, nor are they recommended by the guidelines for the treatment of diseases (Han et al., 2016).

Increased spending on drugs is a global concern (Wettermark et al., 2010), and the problem is even more serious in developing countries, including China. Medical expenditures per capita continued to increase at an average annual rate of 16.3% from 2005 to 2014 in China. (National Bureau of Statistics of the People’s Republic of China, 2015). The surging costs of health care in China is strongly related to the high expenses in pharmaceutical costs (Hu et al., 2019). Drug expenditures accounted for 48.3% of total outpatient healthcare expenditures and 38.3% of total inpatient healthcare expenditures in 2014, suggesting that drug expenditures have been extremely high (Li and Yu, 2021). These proportions are among the highest in the world, compared to an averages determined by the Organization for Economic Co-operation and Development (OECD) of around 17% (OECD Library, 2013; Zhang et al., 2015). A deficiency of the approval of new drugs by the China Food and Drug Administration and the profit incentives in prescribing have become the main reason for the unreasonable use of adjuvant drugs in China (Zhong et al., 2016; Li et al., 2012; Zhang et al., 2015). The current excessive use of Key Monitoring Drugs not only leads to an increased incidence of adverse drug reactions and health issues, but it can also contribute to serious financial burden for patients, result in a wastage of medical resources, and place significant undue pressure on the medical insurance fund (Yang J. et al., 2021a).

The management of Key Monitoring Drugs has been one of the primary methods implemented in China to control the rapid growth of pharmaceutical expenditures (Liang et al., 2018), and since 2015 the Chinese health administrative authorities have taken a series of actions on the management of Key Monitoring Drugs. In 2015, the National Health and Family Planning Commission of the People’s Republic of China indicated that the catalogue of Key Monitoring Drugs must be properly defined, and a tracking and monitoring system of Key Monitoring Drugs and medicines used in an off-label manner must be established in public hospitals (National Health and Family Planning Commission, 2015). In 2016, the Chinese State Council issued a report on the key tasks of deepening the reform of the medical and health system. The report requires that the irrational prescription of Key Monitoring Drugs and nutritional drugs must be monitored to curb the unreasonable increasing growth of pharmaceutical expenditures initially (General Office of the State Council, 2016). Subsequently, many local Health Commissions established the catalogue of Key Monitoring Drugs, and by 2019, Health Commissions of 14 provinces and 9 municipalities have published these catalogues and promulgated relevant policies designed to restrict or supervise the use of Key Monitoring Drugs. However, due to the unclear definition of Key Monitoring Drugs and the different Key Monitoring Drugs catalogues compiled by local Health Commissions, the effectiveness of the above interventions has been insufficient. Since the catalogue of Key Monitoring Drugs has not been established by the Health Commission of Shaanxi Province, the supervision of Key Monitoring Drugs in Xi’an has had little effect. With a 15% price mark-up on prescribed drugs allowed for public Health Care Institutions in China since the 1980s (Eggleston et al., 2008), significant incentives for profit-making activities and the resulting excessive treatment and over prescriptions were recognized as one of the influential factors of the drastic increase in drug expenditures (Mao et al., 2015). Removing the previously allowed 15% profit margin on drugs under the Zero-Mark-Up Drug Policy (ZMDP) severed the link between drug sales and hospital profits. As one of the matching policies of healthcare reform in China, the ZMDP was implemented in Shaanxi province in 2017. However, the ZMDP has not made substantial progress on drug-related expenditures and rational drug use in public hospitals of Shaanxi province (Yan et al., 2020).

To alleviate patients’ medical economic burden, future pharmaceutical reform measures must be carried out to control the excessive and unnecessary use of drugs in hospitals (Yan et al., 2020). The first batch of catalogue of National Key Monitoring Drugs (NKMDs) was released by the Medical Administration Bureau of the National Health Commission of the People’s Republic of China in July 2019 (General Office of the National Health Commission, 2019), which means special rectification targeted at Key Monitoring Drugs for improvement has been scheduled in a national stewardship campaign, and a total of 20 drugs were included (Table 1). Traditionally, the difficulty in management of Key Monitoring Drugs laid mainly in the lack of clear definition of these drugs and the lack of unified supervision methods (Zhong et al., 2016). For the first time, the catalogue of Key Monitoring Drugs were released at the national level, which pointed out the management direction of these drugs. The specific measures recommended in the stewardship policy have become the basis of management. Hence, we derive the testable hypothesis that the consumption of NKMDs will decrease after the implementation of policy interventions.

**TABLE 1:** The catalogue of National Key Monitoring Drugs.

No.	Name	Strengths
1	Monosialotetrahexosylganglioside Sodium Injection^a^	2 ml: 20 mg
2	Cattle Encephalon Glycoside and Ignotin Injection	2 ml, 10 ml
3	Oxiracetam Injection	5 ml: 1 g
4	Creatine Phosphate Sodium for Injection	0.5g, 1 g
5	Deproteinised Calf Blood Serum Injection^a^	0.4 g: 10 ml, 0.2 g:5 ml
6	Alprostadil Injection^a^	2 ml: 10 ug, 1 ml: 5 ug
7	Troxerutin and Cerebroprotein Hydrolysate Injection	2, 5, and 10 ml
8	Coenzyme Complex for Injection	0.1 mg, 0.2 mg
9	Salviae Miltiorrhizae and Ligustrazine Hydrochloride Injection^a^	5 ml
10	Invert Sugar and Electrolytes Injection^a^	500 ml, 250 ml
11	Mouse Nerve Growth Factor for Injection^a^	30 ug, 20 ug
12	Thymopetin for Injection	10 mg
13	Ribonucleic Acid for InjectionⅡ	50 mg, 100 mg
14	Edaravone Injection^a^	30 mg: 20 ml, 10 mg: 5 ml
15	Ossotide Injection	2 ml: 10 mg, 10 ml: 50 mg
16	Cerebroprotein Hydrolysate for Injection^a^	60 mg, 30 mg
17	Ribonucleic Acid for Injection	6 mg, 10 mg
18	Vinpocetine for Injection^a^	5 mg
19	Deproteinized Calf Blood Extractives for Injection^a^	400 mg
20	Cinepazide Maleate Injection	2 ml: 80 mg, 10 ml: 320 mg

a Drugs included in our study.

To the best of our knowledge, the effectiveness of the national stewardship policy on NKMDs consumption has not been characterized in Northwestern China. This study was designed as a retrospective observational study to determine the trends in prescribing practice of NKMDs during the years 2014–2021. We use an Interrupted Time Series (ITS) analysis to quantitatively evaluate the impact of a national stewardship policy and clinical pharmacists’ interventions on NKMDs prescribing in a tertiary hospital of Northwestern China, with the goal of providing a basis for future NKMDs stewardship in China.

## Materials and Methods

### Data Source and Study Design

Data were obtained from the Xi’an People’s Hospital (Xi’an Fourth Hospital) hospital information system (HIS) monthly for 8 consecutive years, from January 2014 to June 2021. This tertiary hospital is located in Shaanxi Province of Northwestern China and offers comprehensive medical, teaching, and scientific research capacities. The hospital has around 1,300 beds with an average daily admission rate of approximately 4,000 patients and >78,000 inpatient admissions annually.

Collected data included information on the following variables concerning NKMDs: generic names, drug specifications, manufacturers, units, total doses, and unit prices. All the usage of and spending on thirteen NMKDs available in the hospital were collected thoroughly monthly. Once the drugs were prescribed in the hospital, the quantity and spending data were captured and extracted by HIS. Since the consumption and spending of each drug were counted monthly separately. Therefore, there was no recall bias or misclassification bias to contend with. Sample size calculation wasn’t required. Three drugs (Oxiracetam Injection, Cinepazide Maleate Injection, Cattle Encephalon Glycoside and Ignotin Injection) were excluded from this study because they were not used continuously or in small quantities.

Drug usage was defined as DDD (defined daily dose), which is the average maintenance dose per day of a drug when used for its major indication in adults. The DDD of the 10 NMKDs studied here were identified according to the instructions provided by the manufacturer (DDD were not available in the Anatomic Treatment and Chemical classification). The instructions of each of the 10 NMKDs in our study were provided as a supplementary file (see Supplement S1). To evaluate the effect of the policy, consumption related indicators were selected as the main indicators in our study. We chose two parameters to reflect the trends and the changing process of 10 NMKDs consumption: DDDs and spending. DDDs of these drugs were used to assess their rate of consumption. Monthly drug spending was recorded in the Chinese currency Renminbi “yuan” (CNY) and then converted into USD (6.44 CNY equals to 1 USD), and is the total amount spent on each drug, for each month. The total DDDs for each month was calculated by summing the individual DDDs for each of the study NKMDs. Similarly, the total monthly expenditure on NKMDs was calculate as the sum of spending on all individual NKMDs each calculated month.

As this was a nationwide stewardship program and randomization with a control group without the intervention was impossible, an ITS analysis was used for this data (Linden, 2015). To evaluate the effect of the NKMDs policy intervention, we conducted an ITS analysis that lasted for 90 months: 66 months before the intervention (pre-implementation, January 2014–June 2019), 5 months lag in the effect of intervention (July 2019–November 2019), implementation completely in December 2019, and 19 months after the intervention (December 2019–June 2021). The ITS regression was performed with Newey-West standard errors (itsa command in Stata) and all models were investigated for autocorrelation using the Cumby-Huizinga test for autocorrelation (Cumby and Huizinga, 1992) using the actest command in Stata version 14, in which all statistical analyses were performed.

To further investigate the differences in total DDDs and expenditure between the pre- and post-policy implementation periods, the pre-policy trend was extrapolated by 19 months (the total number of additional observed months in the post-policy period) using the tsappend function, followed by the extrapolation of the data based on the linear regression trend using the xi:reg function. The difference between the extrapolated data and the observed data was then calculated, in both absolute and relative terms, at 12-months and 19-months post-implementation. This calculation indicates the difference in the observed data compared to what the expected trend in the data would have produced, had no intervention been implemented.

### Intervention

The catalogue of Key Monitoring Drugs (the same as the national advisory) was released by the Health Commission of Shaanxi Province in September 2019. Based on the relevant policies to restrict and supervise patterns of NKMDs, the catalogue of NKMDs was published and multidimensional interventions measures were formulated in November 2019 at the study hospital. A scientific management system for NKMDs was established through administrative intervention, educational programs, prescription review and audit, and information management. Medication guidelines were formulated to clearly specify the conditions and principles of clinical application of NKMDs. Several measures were undertaken to ensure that interventions were strictly enforced, including checking the training attendance records, inspecting the prescription audit results, and verifying the bonus and punishment lists. Furthermore, the results of prescription audits were ranked monthly and were closely related to clinicians’ performance pay. The management leading group also checked and monitored the progress of the interventions monthly. The detailed interventions are further described in the Supplement S2. Multidimensional interventions measures were implemented constantly by clinical pharmacists from December 2019 to June 2021.

## Results

Overall, the total combined DDDs for all NKMDs trended downward in the post-intervention period, at a rate of 430.73 DDDs per month (*p* < 0.001), compared to the pre-intervention period. Similarly, the total monthly spending on NKMDs in the post-intervention period was in significant decline at a rate of $4,681.93 USD per month (*p* < 0.001) compared to the pre-intervention period. Based on these results, the policy intervention led to a dramatic 76.23 and 69.70% decrease in total DDDs and spending, respectively, of NMKDs in June 2021 compared with November 2019.

Of the ten NKMDs investigated in this study, six showed a significant (*p* < 0.05) decrease in monthly DDDs following implementation of new national policy, with one (Invert Sugar and Electrolytes Injection) showing a significant increase in monthly DDDs. The remaining three NKMDs (Monosialotetrahexosylganglioside Sodium Injection, Alprostadil Injection and Deproteinized Calf Blood Extractives for Injection) had no significant change in trend of DDDs in the post-implementation months. Similarly, there was a significant decrease in monthly spending on six NKMDs in the post-implementation period, a significant increase in spending for two NKMDs in that period (Invert Sugar and Electrolytes Injection, Deproteinized Calf Blood Extractives for Injection), and two (Monosialotetrahexosylganglioside Sodium Injection and Alprostadil Injection) of which showed no significant change in spending trend in the post-implementation period.

**FIGURE 1:**
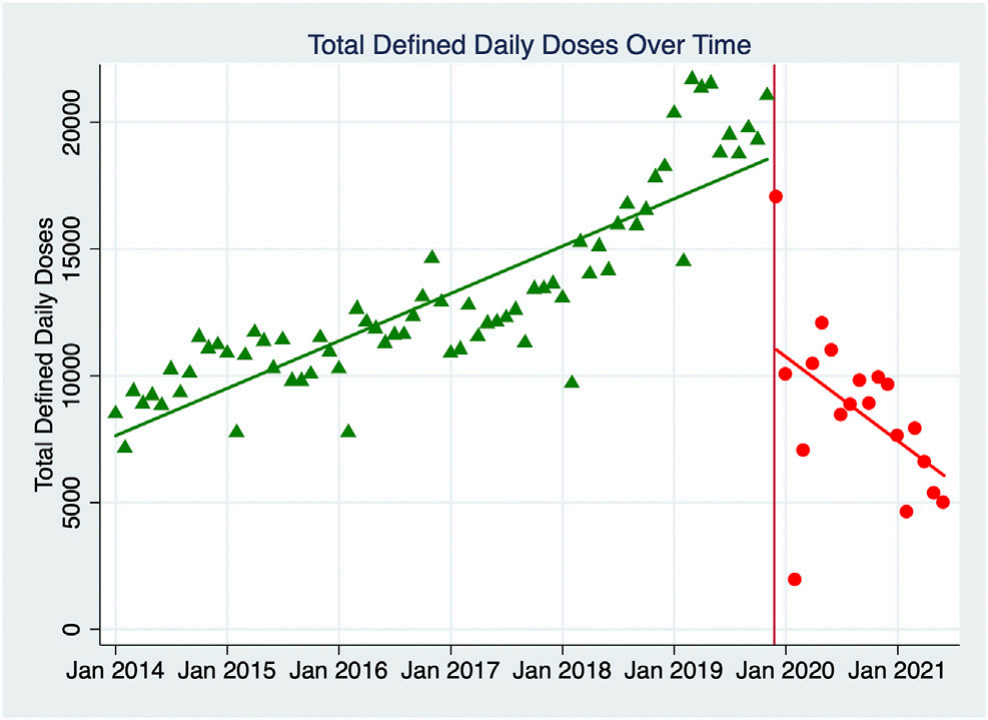
Monthly total Defined Daily Doses of ten NKMDs combined. The monthly DDDs for all NKMDs combined over time was demonstrated, from January 2014 to June 2021. The linear trends of DDDs in the pre-intervention period and the post-intervention period were labeled with different colors, green-pre-intervention period and red-the post-intervention period.

**FIGURE 2:**
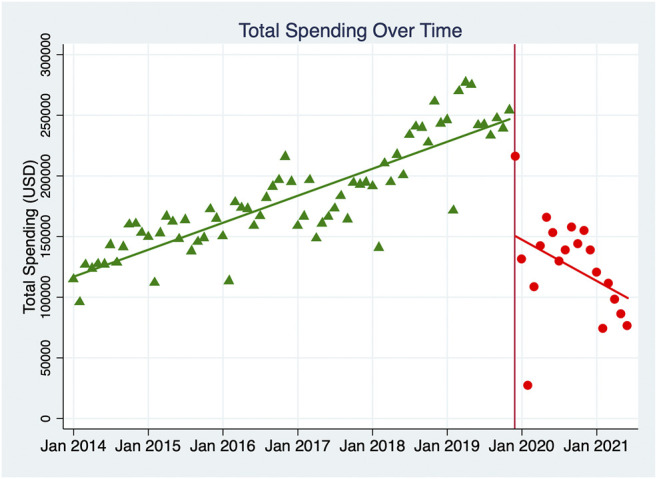
Monthly total spending (USD) of ten NKMDs combined. The monthly spending (USD) for all NKMDs combined over time was demonstrated, from January 2014 to June 2021. The linear trends of spending (USD) in the pre-intervention period and the post-intervention period were labeled with different colors, green-pre-intervention period and red-the post-intervention period.

Table 2 shows the results of the ITS analyses for total DDDs and total spending, indicating the original starting value, the trend in values in the pre-implementation period, the value during the month of implementation, and the trend in values in the post-implementation period. Post-trend analyses for both 12 and 19 months post-intervention were completed, and the comparisons between the observed data and those predictions were calculated, which indicated the intervention effect was effective and sustained. Figure 1 demonstrates the monthly DDDs for all NKMDs combined over time, from January 2014 to June 2021, and indicates the linear trend of DDDs in the pre-intervention period and the post-intervention period. Figure 2 displays similar data for total combined spending on NKMDs over time. Supplementary Table S1 and Supplementary Figure S1 shows individual ITS analyses for DDDs of all ten NKMDs (Supplementary Table S1 and Figure 1), and Supplementary Table S2 and Supplementary Figure S2 shows individual ITS analyses for spending on all ten NKMDs (Supplementary Table S2 and Figure 2).

**TABLE 2:** Interrupted Time Series analyses for total DDDs and total spending.

	Total DDDs	Total spending
Trend Prior to Policy (95% CI)	155.78	1,855.34
	(117.48–194.08)	(1,564.32–2,146.36)
December 2019 - Immediate Change (95% CI)	−7,674.73	−98,363.65
	(−10,675.96–4,673.50)	(−138,435.20–58,292.15)
Trend December 2019 - June 2021	−430.73	−4,681.93
	(−634.90–226.57)	(−7,721.50–1,642.36)
Constant (95% CI)	7,475.65	114,990.40
	(6,266.81–8,684.48)	(105,431.40–124,549.30)
12 Months Extrapolated Difference^a^	−10,463.71	−114,280.09
12 Months Relative Extrapolated Difference^b^	48.72%	57.51%
19 Months Extrapolated Difference	−18,369.70	−228,073.50
19 Months Relative Extrapolated Difference	21.38%	25.03%

a Difference is calculated as the actual observed value of total DDDs or total spending at 12- or 19-months post-intervention, minus the extrapolated value of total DDDs or total spending at 12- or 19-months post-intervention, following the trend from January 2014 to November 2019.

b Relative difference is calculated as the actual observed value of total DDD or total spending at 12- or 19-months post-intervention, divided by the extrapolated value of total DDDs or total spending at 12- or 19-months post-intervention, following the trend from total DDDs January 2014 to November 2019 total spending.

## Discussion

This study evaluated the change in usage of NMKDs, from the perspective of DDDs and spending, after a strict national stewardship policy was implemented. This study revealed that there was an increasing trend in the usage and spending of the ten NKMDs between 2014 and 2019, but that this significantly decreased after the introduction of the multidimensional intervention managed by clinical pharmacists under the implementation of the national stewardship policy. While the overall use pattern of NKMDs within seasons was similar, the intervention led to a dramatic 76.2 and 69.7% decrease in DDDs and spending of NMKD, respectively, in June 2021 compared with November 2019, which reflects the national government-driven policy could be considered an effective strategy. In April 2019, the National Health Commission of the People’s Republic of China issued a notice on drug use monitoring and clinical evaluation, which requires a comprehensive evaluation of drug utilization in order to improve local medical support systems and the quality of treatment services (National Health Commission, 2019). Clinicians have an obligation to ensure that the medicines they prescribe do not result in increased harm or cost unless there is at least a reasonable expectation of a benefit to the patient. Thus, the most rational route toward correct use of NKMDs is to prescribe them by strictly adhering to evidence-based guidelines. Therefore, clinical practice guidelines and recommendations for clinical application of NKMDs was published to ensure doctors’ prescribing behavior is suitable to clinical needs. Guidance, education, stewardship, and supervision on rational NKMDs use were addressed as professional strategies together with administrative strategies. At Xi’an People’s Hospital, the interventions were managed by clinical pharmacists and supported by the Pharmaceutical Management Professional Committee, with multiple other sectors participating as well. The strict enforcement of the above stewardship campaign also contributed to the decline in drug usage and medication spending. The interventions were found to be effective in facilitating the safe and rational use of drugs for clinical purposes. Several other studies have also indicated the positive effects of this policy on controlling the growing consumption of NKMDs, promoting the clinical rational use of NKMDs and savings in unnecessary drug expenses for patients (Yang J. et al., 2021a; Ke et al., 2021; Zhang W. et al., 2021b; Wang et al., 2020), which was consistent with our study. A recent study which evaluated the effect of the national stewardship on NKMDs prescribing in the secondary and tertiary hospitals of Guangdong province found that spending on NKMDs as the proportion of total expenditure on medication decreased from 4.8% in the first half of 2018 to 2.7% in the second half of 2019, following policy implementation (Ke et al., 2021). A study indicated that the establishment of clinical rational NMKDs use evaluation system based on the multi-disciplinary collaboration model had led to a significant decrease in irrational prescribing of NKMDs, and in addition the dosage and spending of 8 NKMDs decreased significantly (Zhang W. et al., 2021b). Another study indicated that the proportional expenditure on seven NKMDs decreased from 4.4% of total medication spending to 2.8% and a drop of 25.8% in DDDs of the consumption of NKMDs in a teaching hospital after the interventions (Wang et al., 2020). That overall NKMDs use significantly decreased in February 2020 in our study may be explained in part due to the impact of the outbreak of COVID-19, during which the government advocated for people to stay at home. However, by May 2020, the number of patients visiting the hospital had returned to equal to the same period in 2019.

NKMDs included in the first batch of catalogues were mainly drugs involved in the prevention or treatment of cardiovascular, cerebrovascular and nervous system diseases, tumor adjuvant therapy, and nutritional electrolyte preparation. We observed that the gradual increasing trend, both in monthly DDDs and spending, for 6 of the 10 medications during the pre-implementation period turned into a significant downward trend in the post-implementation period. In contrast, we did not observe a significant decreasing trend for prescriptions of Monosialotetrahexosylganglioside Sodium Injection, Deproteinized Calf Blood Extractives for Injection and Alprostadil Injection in the post-implementation period. The driving force for these differential changes in prescribing trends is unclear. A partial explanation may be that the off label prescribing of Deproteinized Calf Blood Extractives for Injection, Alprostadil Injection, and Monosialotetrahexosylganglioside Sodium Injection in ophthalmology has not been significantly changed in our hospital. A previous study found that the proportion of the purchase expenditure of Monosialotetrahexosylganglioside Sodium Injection showed a significant downward trend (Ke et al., 2021), which wasn’t consistent with our study. There was a significant increased trend both in monthly DDDs and spending on Invert Sugar and Electrolytes Injection in the post-implementation period. Invert Sugar and Electrolytes Injection was remain widely used in the department of otolaryngology for postoperative nutritional support treatment for patients undergoing surgery of the pharyngeal region, tonsils and thyroid, as well as patients in emergency department in our hospital after the intervention. However, nutritional support treatment with Invert Sugar and Electrolytes Injection should be limited to critically ill patients (for perioperative patients undergoing major operation or complicated procedures, trauma, tumor, infection, burns, shock, etc.) or patients with a history of diabetes or hyperglycemia, while patients undergoing minor operation without abnormal electrolytes or blood glucose during hospitalization was not recommended (Zhu et al., 2017). This phenomenon highlights the potential for further supervision in these medication through targeted intervention.

The proper use of NKMDs is conducive to the rehabilitation of patients, while over prescription and irrational use of NKMDs not only increases the economic burden of patients and the country’s medical resources, but also increases the risk of adverse drug reactions due to combinations or unnecessary use of drugs. According to the Pharmaceutical Data Base of China’s National Pharmaceutical Industry Information Center, the total sales of the 20 NKMDs covered by the “national policy” exceed $1.55 billion USD in 2018, and the sales of some varieties of NKMDs in hospital exceeded $15.53 million USD (Baidu Research Engines, 2019). In particular, the sales of Oxiracetam Injection totaled nearly $230 million USD, while the sales of Icotinib Hydrochloride Tablets, the first domestic innovative drug in China, was $ 190 million USD which reached a “record high” in 2018 (People’s Daily Online, 2019). Previous studies have shown the documented misuse of NKMDs was common in public hospitals of China (Lin et al., 2015; Zhu et al., 2017; Zhang H. et al., 2021a). The rate of irrational usage of Invert Sugar and Electrolytes Injection was 26.6% in a large tertiary teaching hospital in January 2016, which were comprised of unapproved indications, overly long treatment durations, contraindication, and inappropriate drug combinations (Zhu et al., 2017). Another drug-utilization study showed that Salviae Miltiorrhizae and Ligustrazine Hydrochloride Injection was used off-label extensively in malignant tumor patients, which accounted for 11.76% of the total 38,126 patients from 24 tertiary hospitals (Zhang H. et al., 2021a). This phenomenon has since led to serious concerns from the public about the safety and quality of health care (Han et al., 2016). To reduce the risk of medication and strengthen drug use monitoring, China’s Center for Drug Evaluation of National Medical Products Administration issued a notice on suggestions for revision of the instructions for Invert Sugar Injection and Invert Sugar and Electrolytes Injection in June 2018, which removed the recommended use of these drugs as diluents under the indications, and added adverse drug reactions involving digestive, respiratory, nervous, circulatory, urinary system diseases, skin and subcutaneous tissue, eye diseases and local reactions (China’s Center for Drug Evaluation of National Medical Products Administration, 2018). According to a document issued by China’s Center for Drug Reevaluation of National Medical Products Administration (China’s Center for Drug Reevaluation of National Medical Products Administration. 2016), the drug instruction of Monosialotetrahexosylganglioside Sodium Injection was revised to include warning words, ADR information, contraindications, and usage in children. China’s Center for Drug Reevaluation of National Medical Products Administration issued notice on the revision of the instructions for Salviae Miltiorrhizae and Ligustrazine Hydrochloride Injection in September 2019 (China’s Center for Drug Reevaluation of National Medical Products Administration, 2019), and ADR information of the risk of severe allergic reactions especially anaphylactic shock, contraindications and warnings were added.

In recent years, implementing health insurance payment methods that encourage the physician to deliver cost-effective health services represent a promising direction in China’s new healthcare reform. Policy makers are exploring to expand medical payment systems of Global Budget Payment System (GBPS) and Diagnosis Related Group System (DRGs) to curb the excessive growth of medical expenditure (Huang et al., 2016; Liu et al., 2017). These systems offer a prospective reimbursement to hospitals, with the total expected spending determined ahead of a budget year mainly based on a fee-for-service or grouping of patients according to their diagnosis and other traits. The DRG system is a payment mechanism known as the “ceiling price for a single disease”, which forces the control of medical expenditures and promotes the efficient utilization of medical resources in hospitals. The shift from the traditional retrospective cost-based system to prospective DRG-based system led to the containment of medical costs (Liu et al., 2017). One study has indicated that the proportion of expenses on Key Monitoring Drugs of total drugs was significantly decreased, which provided a reference for continuous optimization of the expense management model after management of Key Monitoring Drugs based on DRGs (Yang X. et al., 2021b). In June 2019, National Healthcare Security Administration issued a notice on the requirement of attempted implementation of the medical payment system for DRGs in 30 pilot cities (National Healthcare Security Administration, 2019). DRGs were implemented in four hospitals of Xi’an in July 2021, including Xi’an People’s Hospital. In August 2021, the National Health Commission of the People’s Republic of China, (2021) issued documents requesting the hospitals to adjust the catalogues of NKMDs. The catalogues of NKMDs were requested to include 30 drugs selected from six categories of drugs (including Adjuvant Drugs, PPIs, antibacterials, etc.) because of abnormally large consumption and the current situation of irrational use in hospitals (National Health Commission. 2021). This means that the subsequent catalogues of NKMDs will continue to be updated, and the dynamic management of NKMDs will be established moving forward. While challenges remain, the Chinese government has focused on the appropriate use of NKMDs and the impact of the catalogue will be sustained.

This study has three main strengths. First, this was the first quantitative study to investigate the impact of the national stewardship on the trends of NKMDs prescribing pattern in hospital of the Northwestern China (DDDs and spending were selected as the main indicators). Second, the availability of longitudinal data from the hospital allowed examination of long-term associations between a stewardship intervention and NMKDs prescribing. There were no significant changes to the organization of the hospital during the study period, which allowed for a stable population over the study period. Furthermore, the analyzed data were captured routinely, meaning the likelihood of missing data is very low. Finally, the ITS analysis used is a robust design for evaluation of real-world interventions which cannot be randomized, and there were adequate time points before and after the intervention in our data series.

Our study has several limitations. First, it was conducted in Xi’an only, which may not represent the general situation in China. Despite social and economic conditions and doctors’ prescription behavior differing across regions in China, the NKMDs stewardship program was carried out at the national level. Our study serves as a practical case to illustrate that NKMDs abuse can be effectively controlled through scientific management. Therefore, the multidimensional interventions embedded in our research for reducing NKMDs prescribing are worthy of reference for other hospitals of China. The second limitation is that comparisons based on the spending parameter may sometimes offer reduced value in the evaluation of drug use. The prices of all NKMDs decreased by 15% under the influence of the ZMDP policy implemented in Shaanxi province in April 2017. And the prices of some varieties of NKMDs decreased slightly under the influence of the National Centralized Drug Procurement (NCDP) implemented in Xi’an in January 2019. Fluctuations in prices of the same preparations between different years may lead to bias and make the long-term evaluation difficult. However, because pricing fluctuations occurred before the intervention policy was put in place in November 2019 and the total spending of 10 NKMDs increased during that period, its influence on the results of this study are limited.

Our study results have important policy implications. At present, the national policy tends to take the overall consumption and spending change as the indicators of management effect. However, there are potential limitations if only the declining rate of consumption is used as the indicator in evaluating the utilization of Key Monitoring Drugs, nor is it helpful to guide rational drug use in clinical application. Further, indicators are required to comprehensively evaluate the frequency and structure of Key Monitoring Drugs prescribing pattern in hospitals (Wang et al., 2021). On the other hand, the main purpose of the stewardship of Key Monitoring Drugs is to improve rationality of drug use. Considering most secondary or tertiary hospitals have implemented a pre-prescription review system (Liu et al., 2021), this review software can be deployed as a tool to detect, rectify and prevent the occurrence of prescribing problems and improve the quality of pharmaceutical care. After drug-related problems (DRPs) were identified through quick checks of the system, pharmacists reviewed the potentially inappropriate prescriptions. Furthermore, clinical practice guidelines and recommendations for the clinical application of Key Monitoring Drugs should be established as consensus statements at the national scale, which could help achieve the ultimate goal of rational utilization of NKMDs.

## Conclusion

This study evaluated the impact of a national stewardship policy on NKMDs use in a tertiary teaching hospital in the Northwestern China. An interrupted time series analysis showed a decrease in consumption quantity and spending of NKMDs in the study hospital following implementation of the national policy, which indicates that the policy could therefore be considered an effective strategy and the intervention measures were successful. Additional comprehensive policies should be introduced to further improve the rational use of NKMDs.

## Data Availability

The original contributions presented in the study are included in the article/Supplementary Material, further inquiries can be directed to the corresponding author.
