# Supplementary material for: The Impact of a National Stewardship Policy on the Usage Patterns of Key Monitoring Drugs in a Tertiary Teaching Hospital: An Interrupted Time Series Analysis
Source: Front Pharmacol. 2022 Feb 18;13:847353. doi: 10.3389/fphar.2022.847353 (PMC8895446; doi:10.3389/fphar.2022.847353)
Supplement: Supplementary file 1 [file Table1.docx]

**TABLE S1** Interrupted Time Series analyses for defined daily doses of ten national key monitoring drugs

| **Drug** | **Trend Prior to Policy** | **95% CI** | **Dec 2019** | **95% CI** | **Trend  Dec 2019 -**  **Jun 2021** | **95% CI** | **Constant** | **95% CI** |
| --- | --- | --- | --- | --- | --- | --- | --- | --- |
| Salviae Miltiorrhizae and  Ligustrazine Hydrochloride Injection | 18.30 | 11.48 - 25.11 | -1,192.36 | -1606.65 - -778.08 | -117.09 | 152.67 - -81.52 | 1,247.30 | 918.41 - 1576.2 |
| Monosialotetrahexosylganglioside  Sodium Injection | 21.74 | 14.43 - 29.04 | -808.36 | -1177.71 - -439.01 | -11.03 | -25.87 - 3.81 | -1.27 | -246.97 - 244.42 |
| Alprostadil Injection | 41.11 | 26.94 - 55.29 | -2,420.90 | 3441.62 - -1400.18 | -93.65 | 191.66 - 4.35 | 456.84 | 71.7 - 985.39 |
| Deproteinised Calf Blood  Serum Injection | 46.26 | 33.11 - 59.42 | -1,405.14 | 2466.24 - -344.04 | -83.35 | 157.94 - -8.76 | 1,001.45 | 482.68 - 1520.21 |
| Edaravone Injection | 1.07 | 0.45 - 2.6 | -171.08 | 244.4 - -97.76 | -7.52 | 11.23 - -3.8 | 266.32 | 193.38 - 339.27 |
| Vinpocetine for Injection | 19.90 | 15.89 - 23.91 | -543.20 | -1172.19 - 85.78 | -51.00 | -93.66 - -8.34 | 818.72 | 702.43 - 935.01 |
| Cerebroprotein Hydrolysate  for Injection | 14.58 | 2.48 - 26.69 | 115.94 | -654.64 - 886.52 | -56.75 | -101.66 - -11.85 | 145.57 | -240.26 - 531.42 |
| Mouse Nerve Growth Factor  for Injection | 22.07 | 19.87 - 24.26 | -438.59 | -854.11 - -23.07 | -46.11 | -81.17 - -11.04 | 327.30 | 250.67 - 403.93 |
| Deproteinized Calf Blood  Extractives for Injection | -3.65 | -4.51 - -2.79 | 44.63 | 2.93 - 86.34 | 0.92 | -1.68 - 3.52 | 276.98 | 228.9 - 325.06 |
| Invert Sugar and Electrolytes  Injection | -25.63 | -35.52 - -15.74 | -855.65 | -1324.11 - -387.18 | 34.88 | 14.78 - 54.98 | 2,936.39 | 2536.9 - 3335.88 |
