# Supplementary material for: The Impact of a National Stewardship Policy on the Usage Patterns of Key Monitoring Drugs in a Tertiary Teaching Hospital: An Interrupted Time Series Analysis
Source: Front Pharmacol. 2022 Feb 18;13:847353. doi: 10.3389/fphar.2022.847353 (PMC8895446; doi:10.3389/fphar.2022.847353)
Supplement: Supplementary file 2 [file DataSheet3.docx]

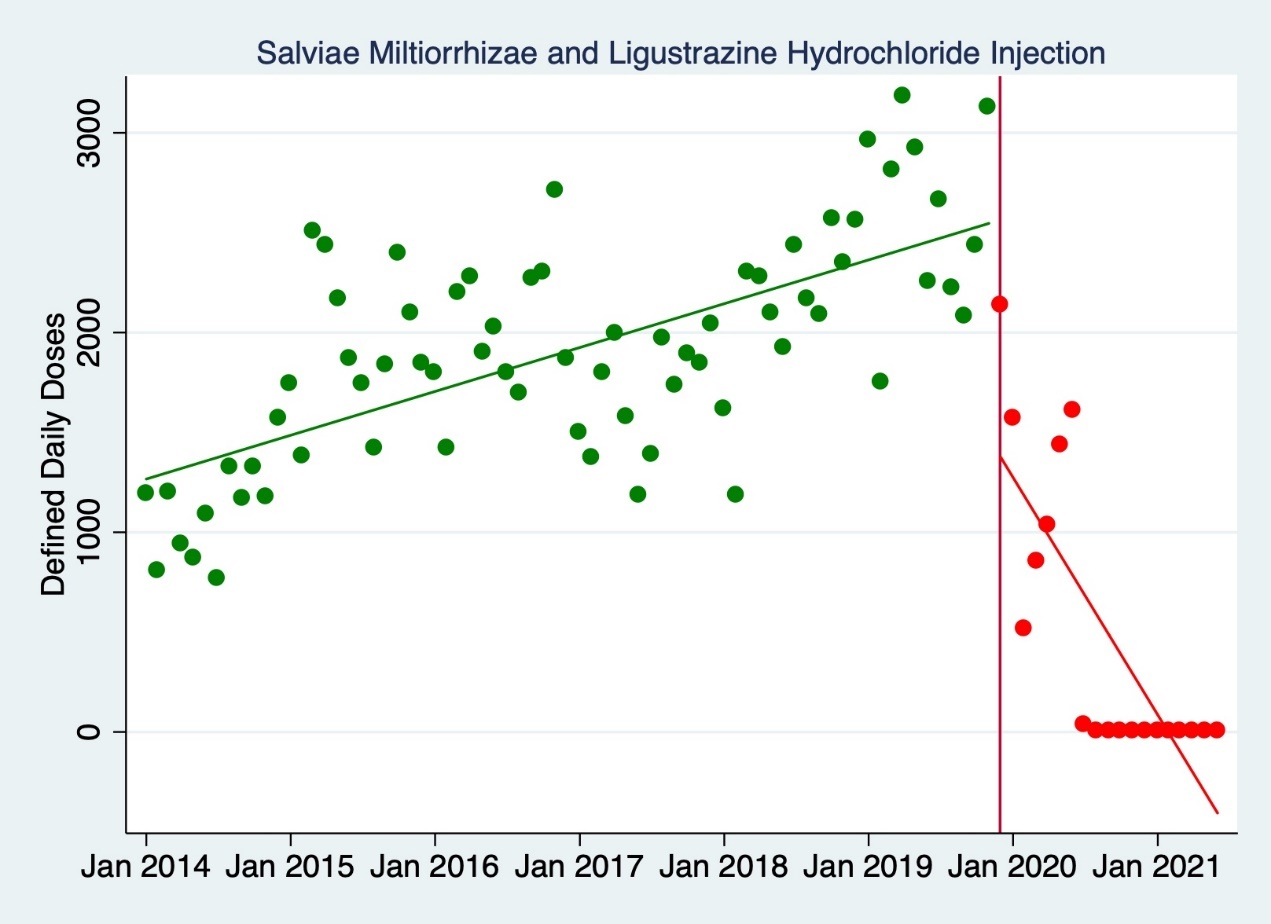


**(A)** Monthly Defined Daily Doses of Salviae Miltiorrhizae and Ligustrazine Hydrochloride Injection


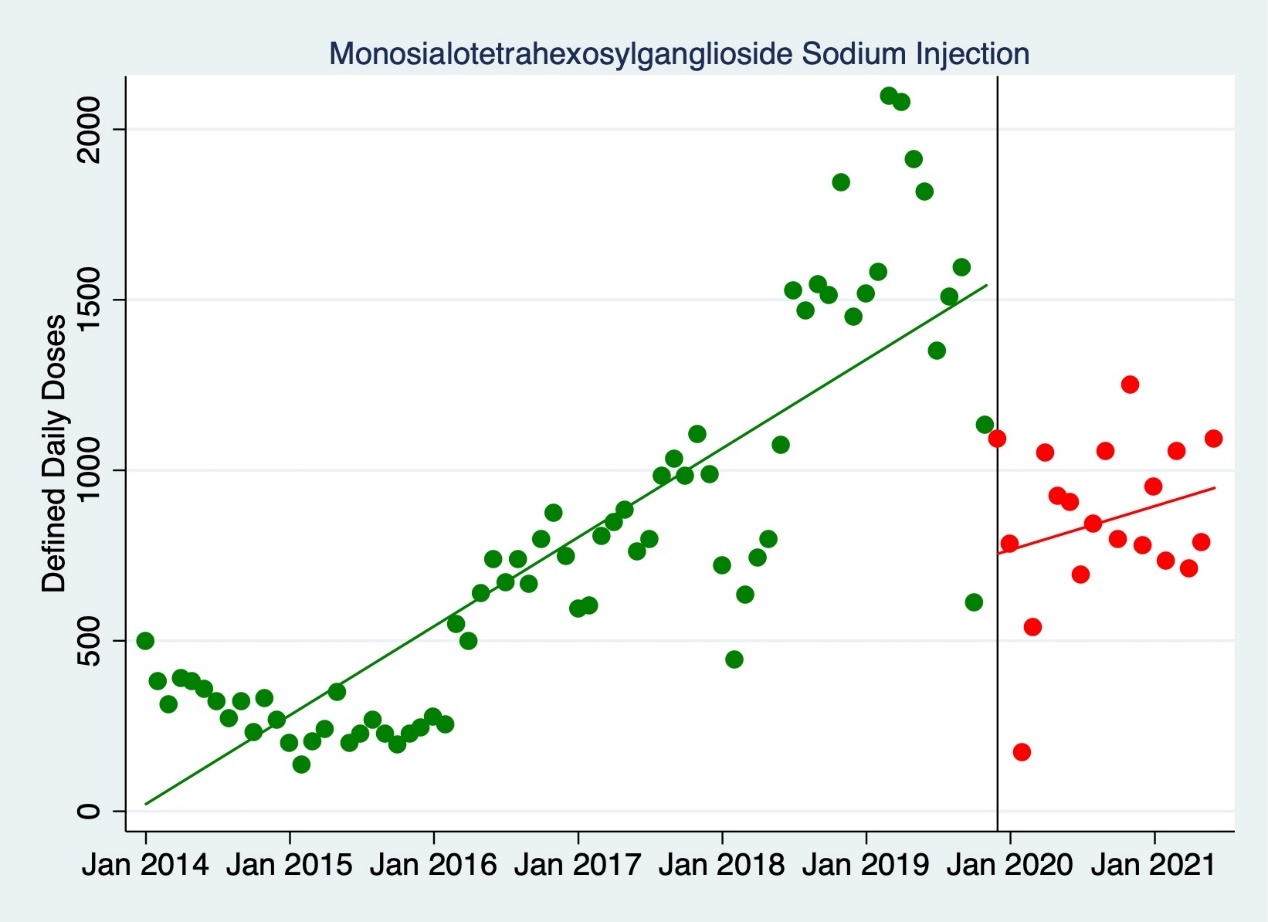


**(B)** Monthly Defined Daily Doses of Monosialotetrahexosylganglioside Sodium Injection


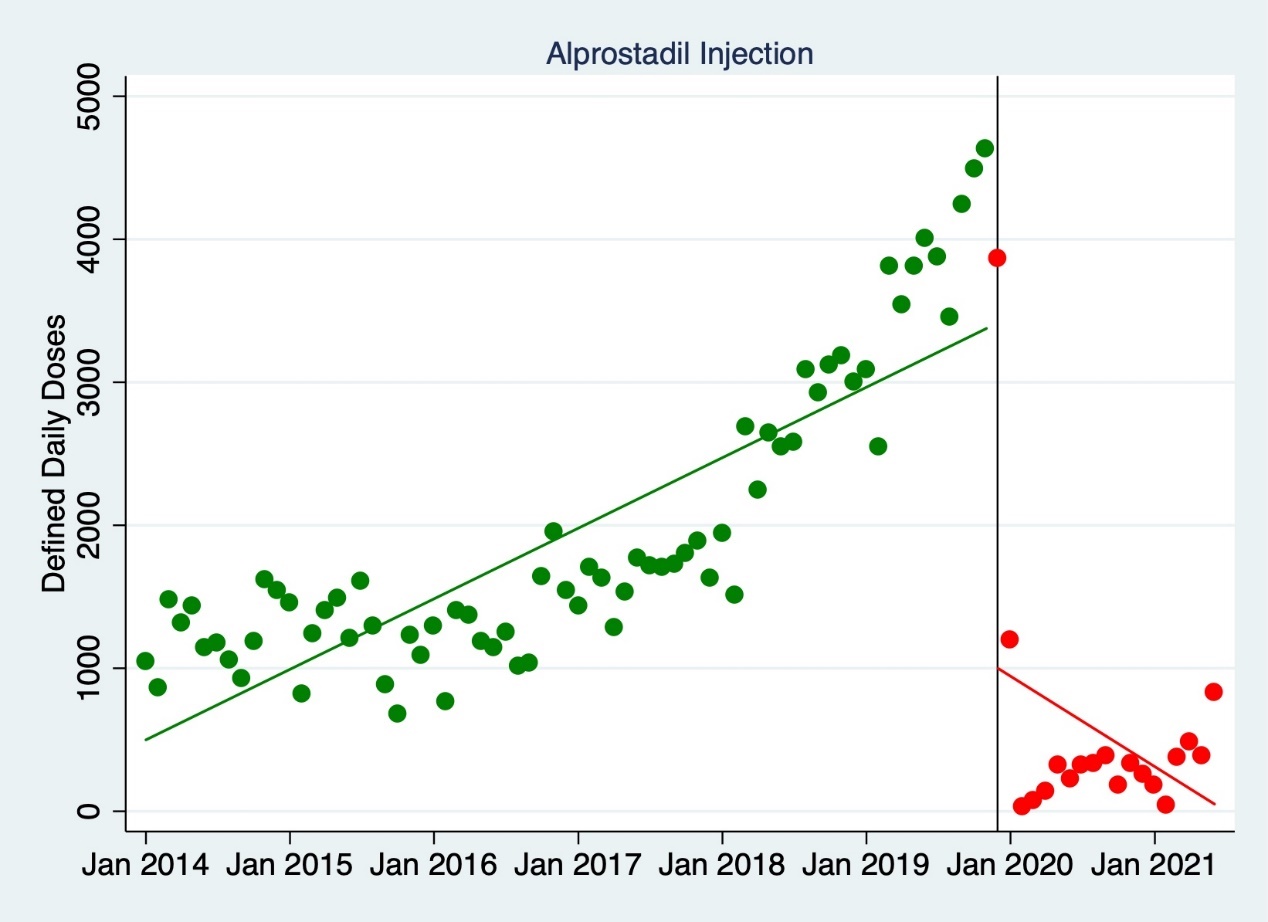


**(C)** Monthly Defined Daily Doses of Alprostadil Injection


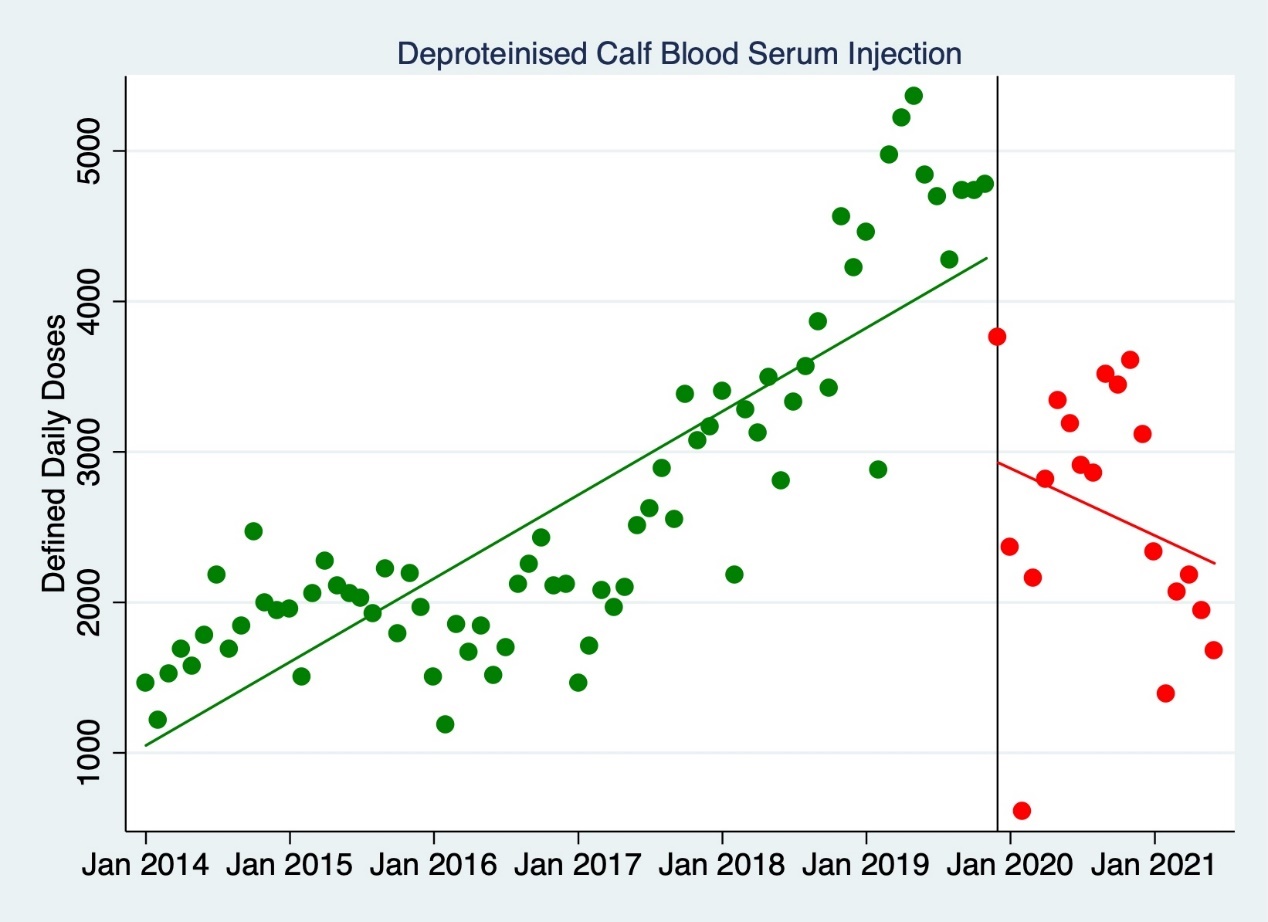


**(D)** Monthly Defined Daily Doses of Deproteinised Calf Blood Serum Injection


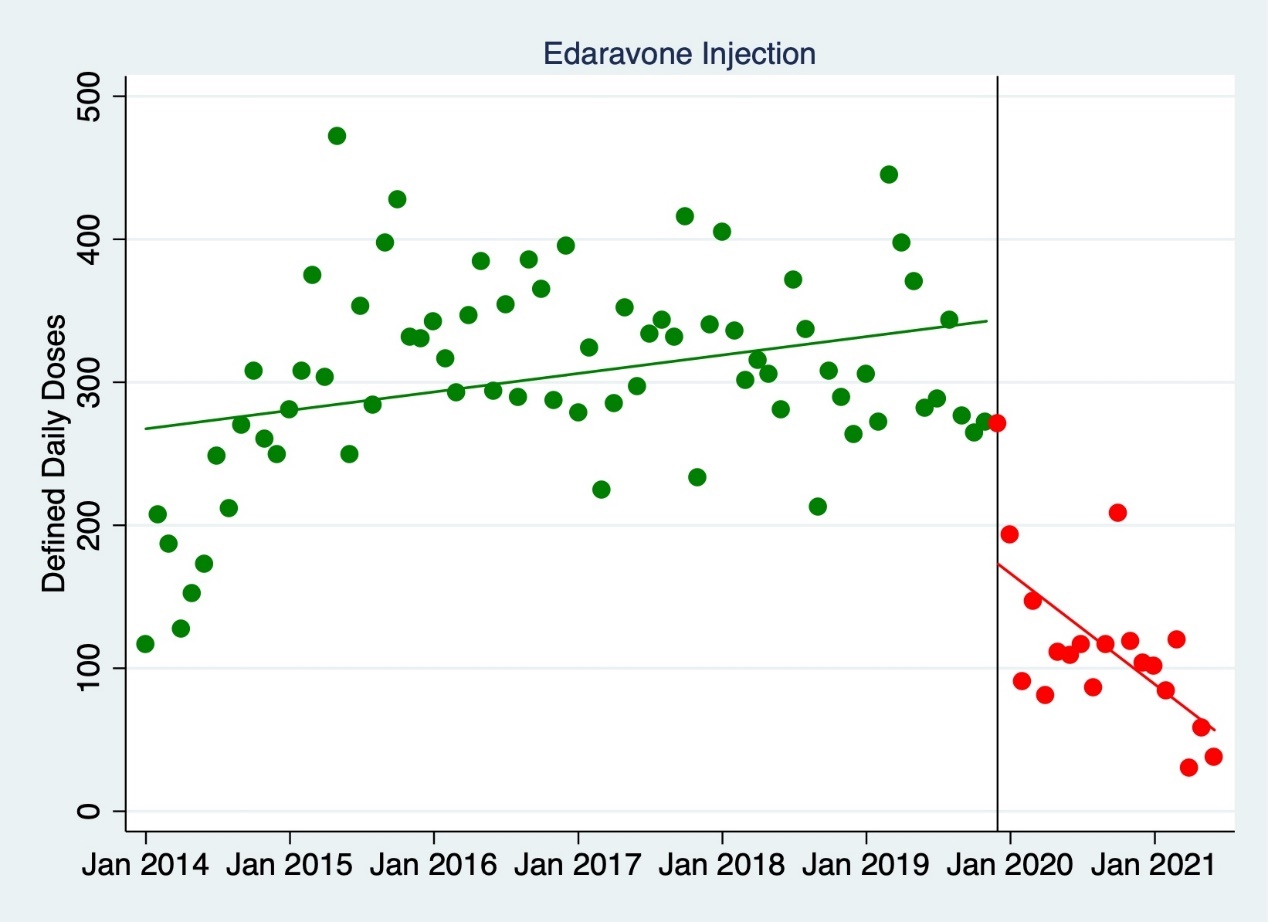


**(E)** Monthly Defined Daily Doses of Edaravone Injection


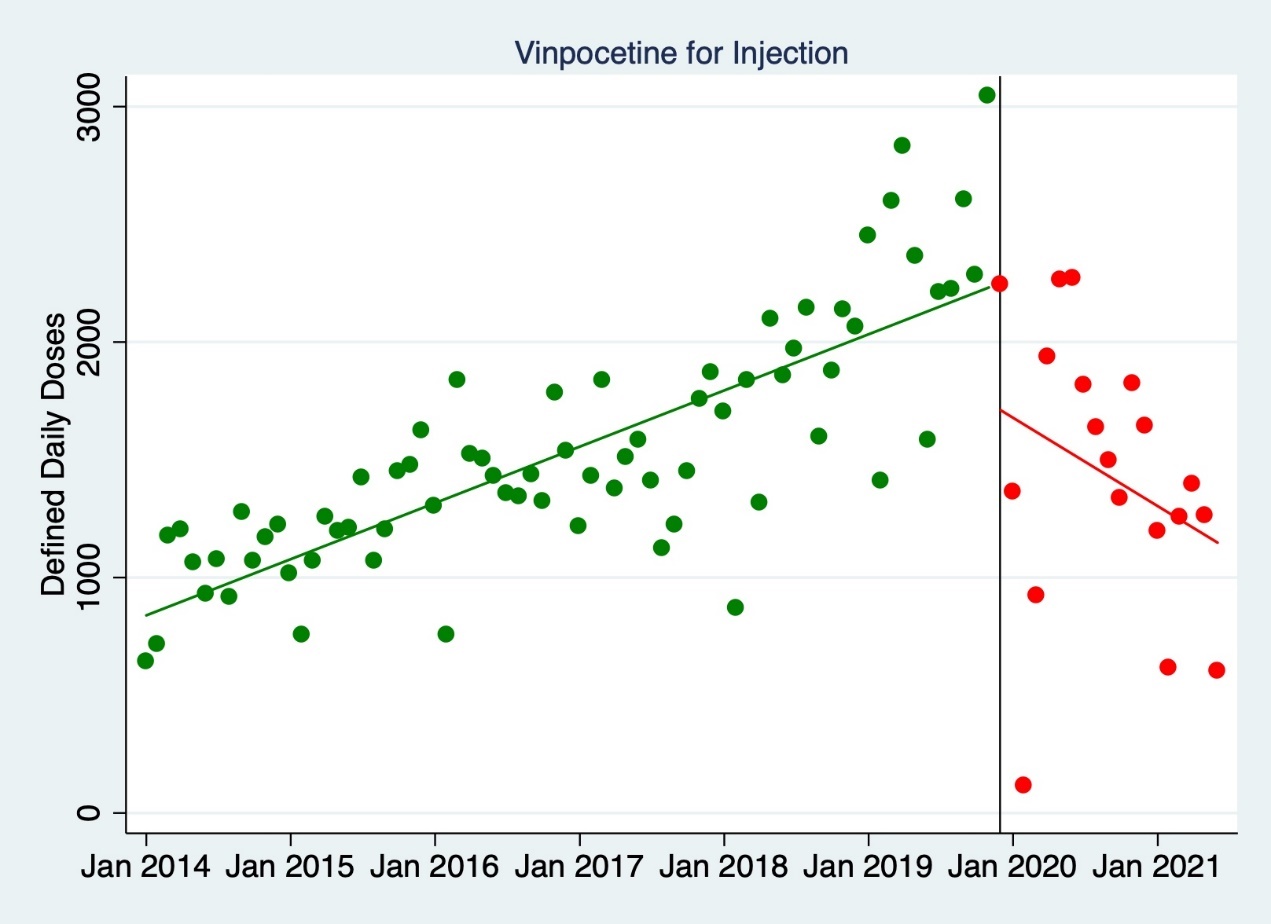


**(F)** Monthly Defined Daily Doses of Vinpocetine for Injection


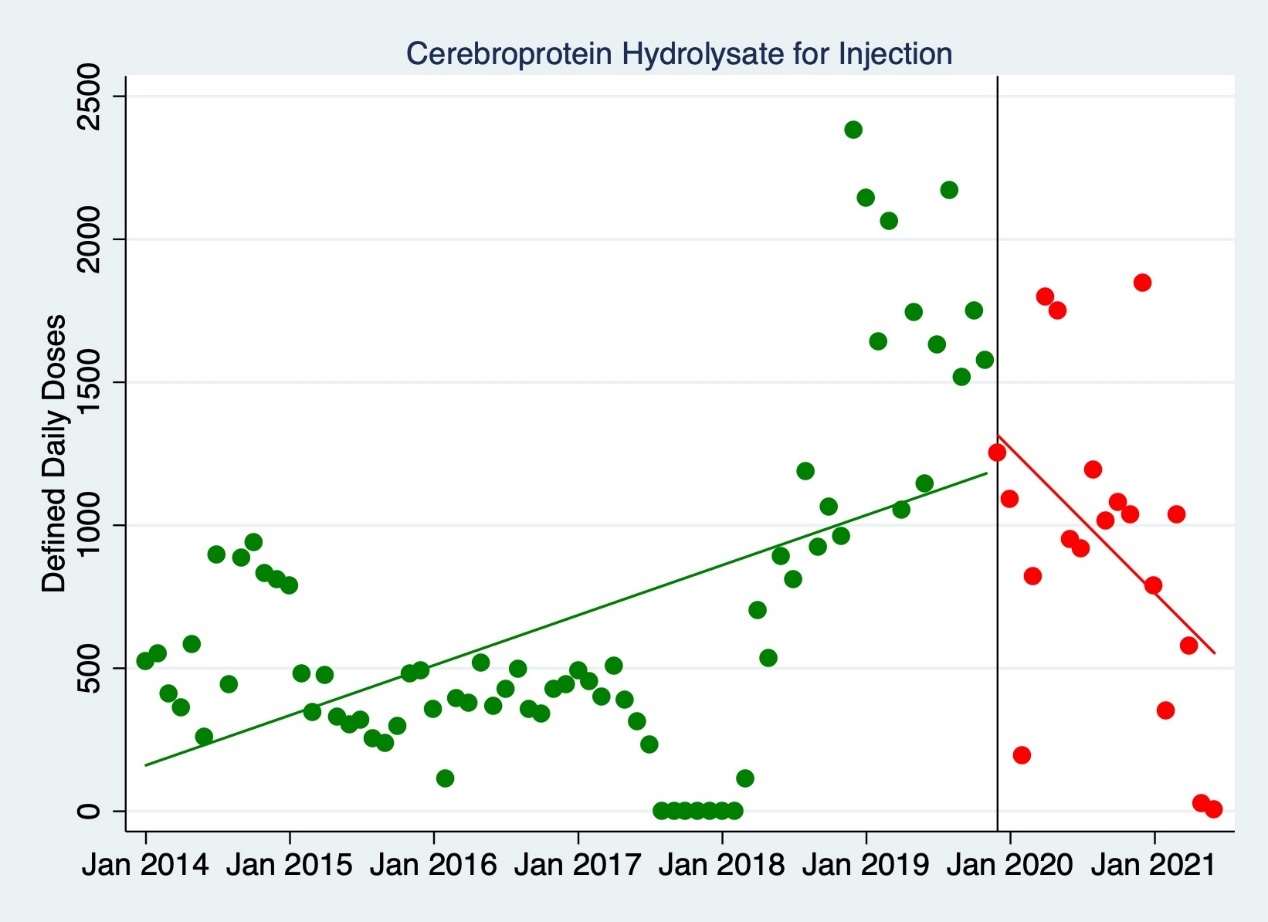


**(G)** Monthly Defined Daily Doses of Cerebroprotein Hydrolysate for Injection


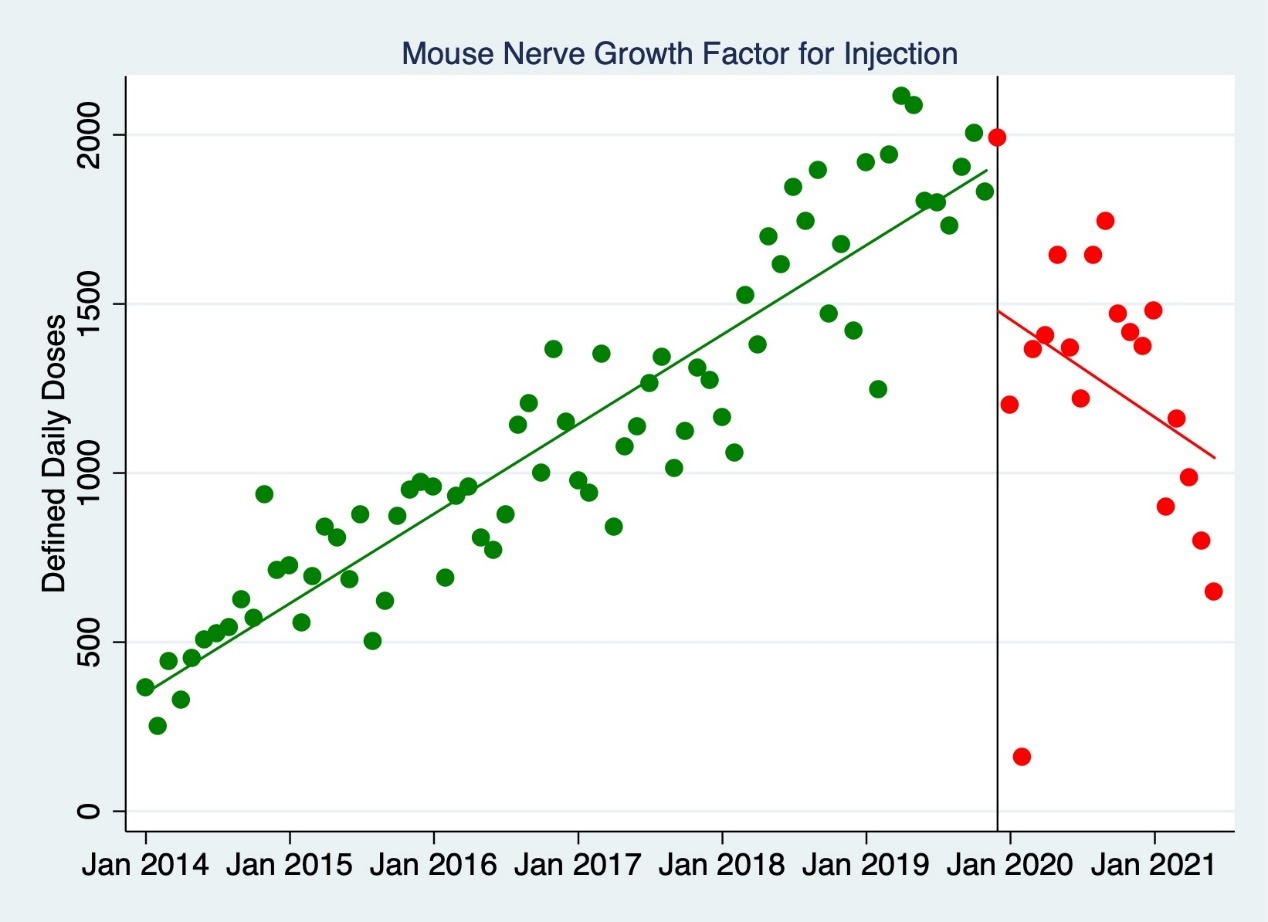


**(H)** Monthly Defined Daily Doses of Mouse Nerve Growth Factor for Injection


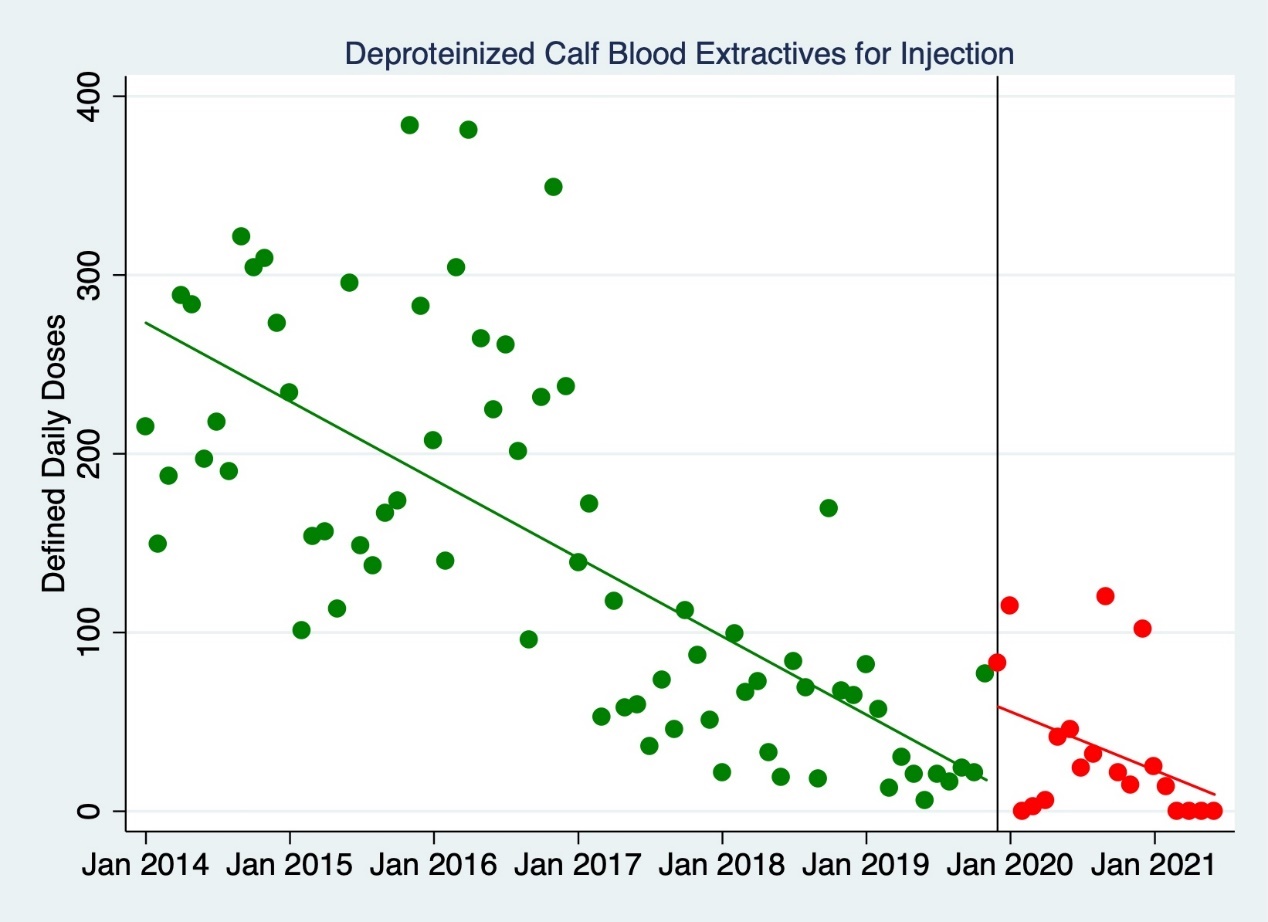


**(I)** Monthly Defined Daily Doses of Deproteinized Calf Blood Extractives for Injection


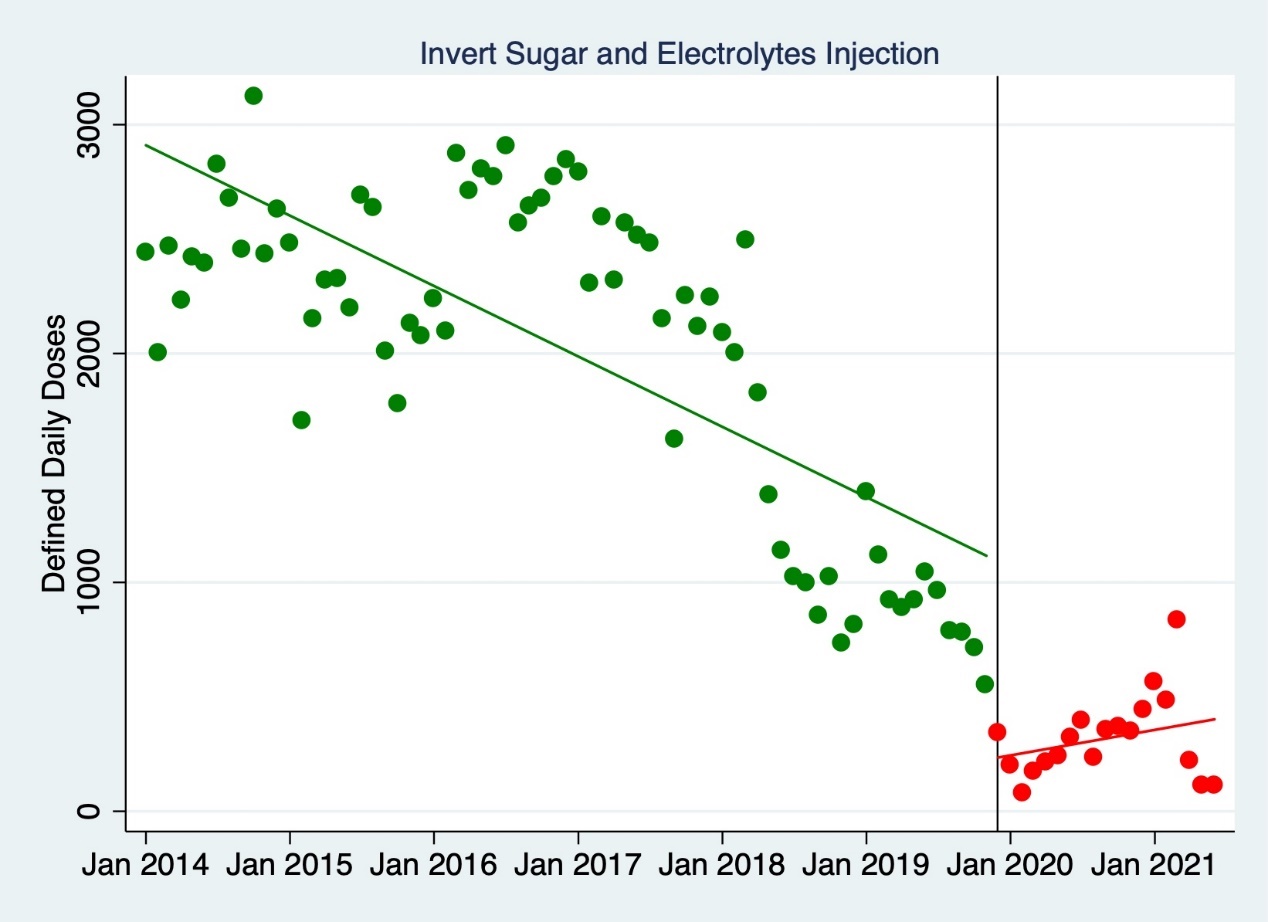


**(J)** Monthly Defined Daily Doses of Invert Sugar and Electrolytes Injection

**FIGURE S1** Interrupted Time Series analyses for DDDs of ten national key monitoring drugs


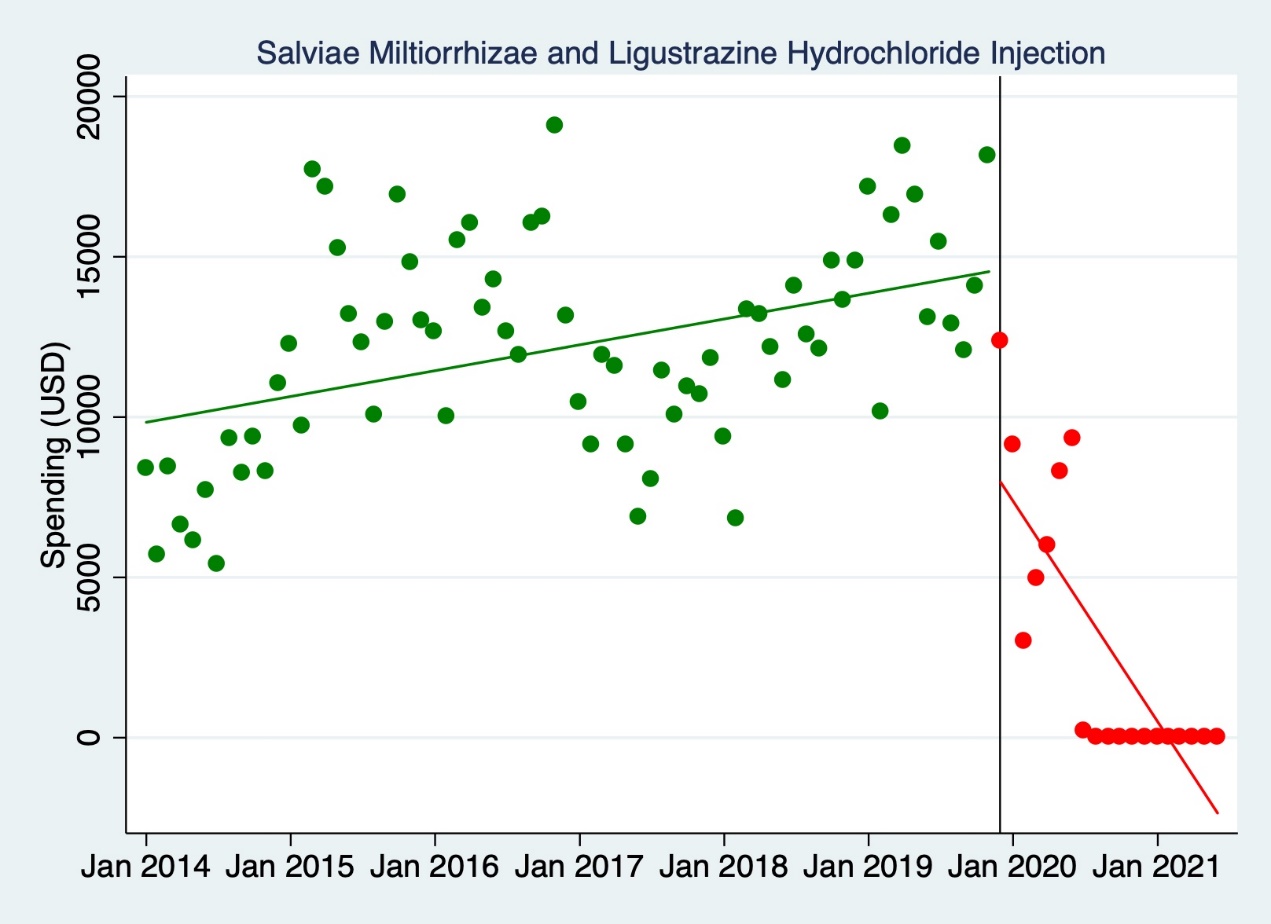


**(A)** Monthly spending of Salviae Miltiorrhizae and Ligustrazine Hydrochloride Injection


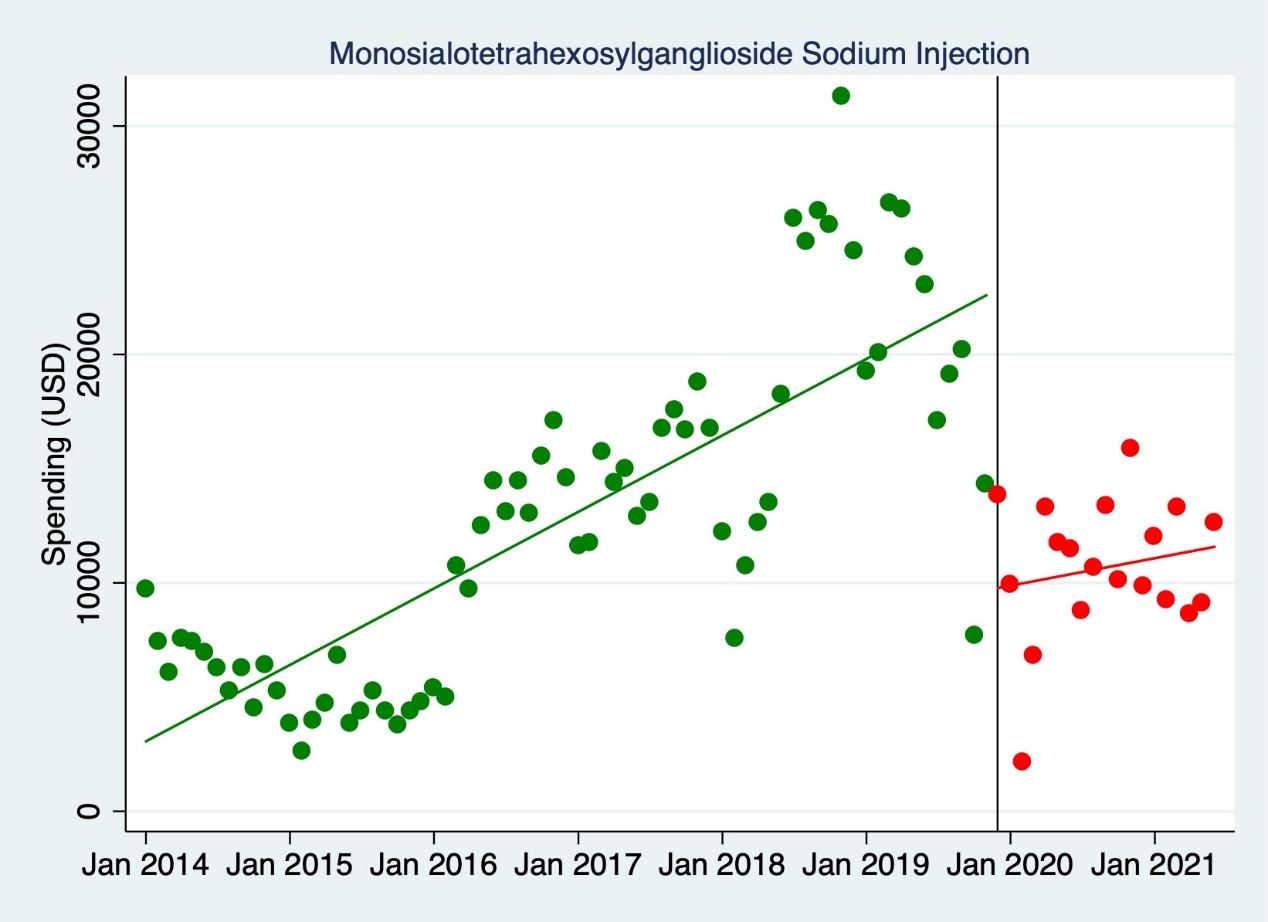


**(B)** Monthly spending of Monosialotetrahexosylganglioside Sodium Injection


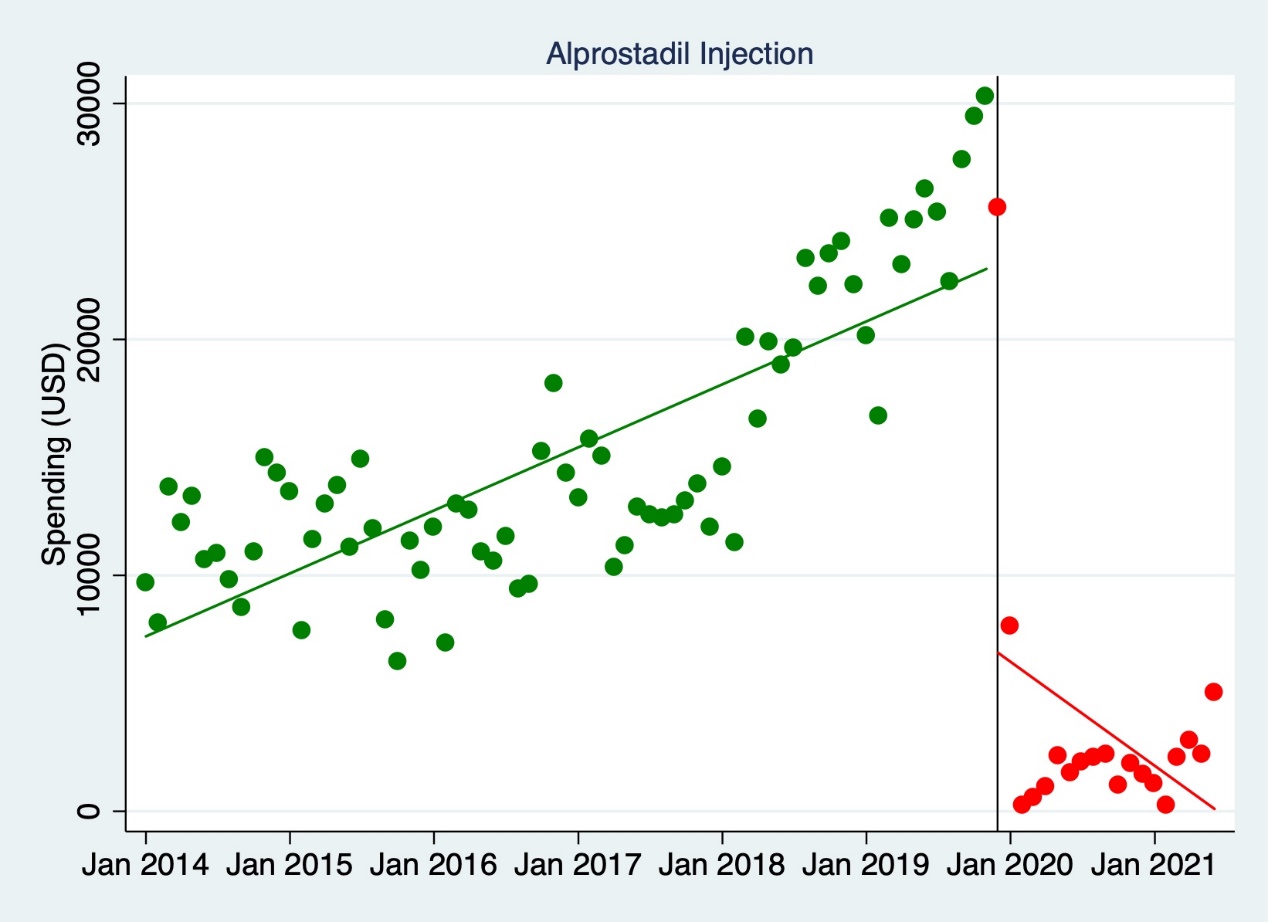


**(C)** Monthly spending of Alprostadil Injection


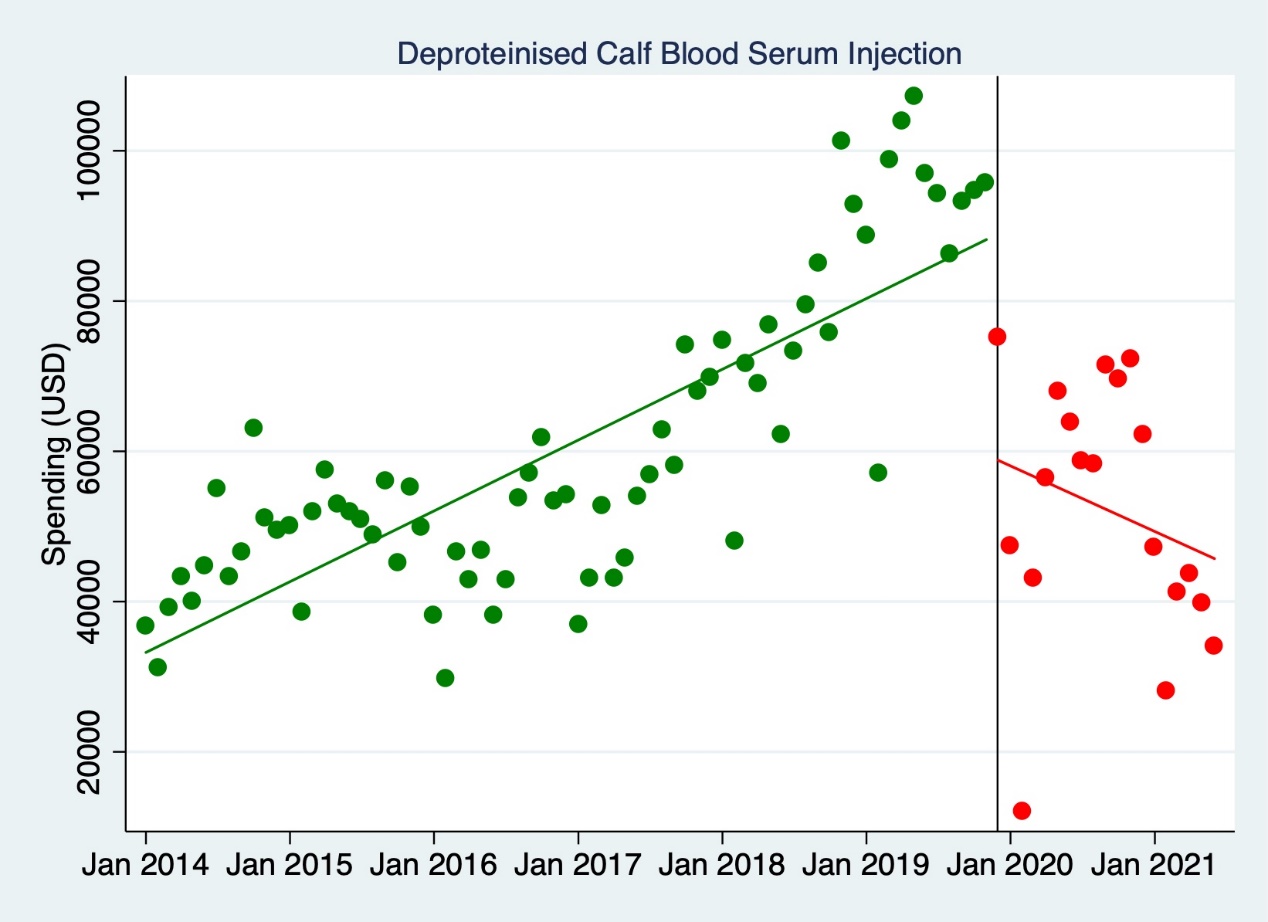


**(D)** Monthly spending of Deproteinised Calf Blood Serum Injection


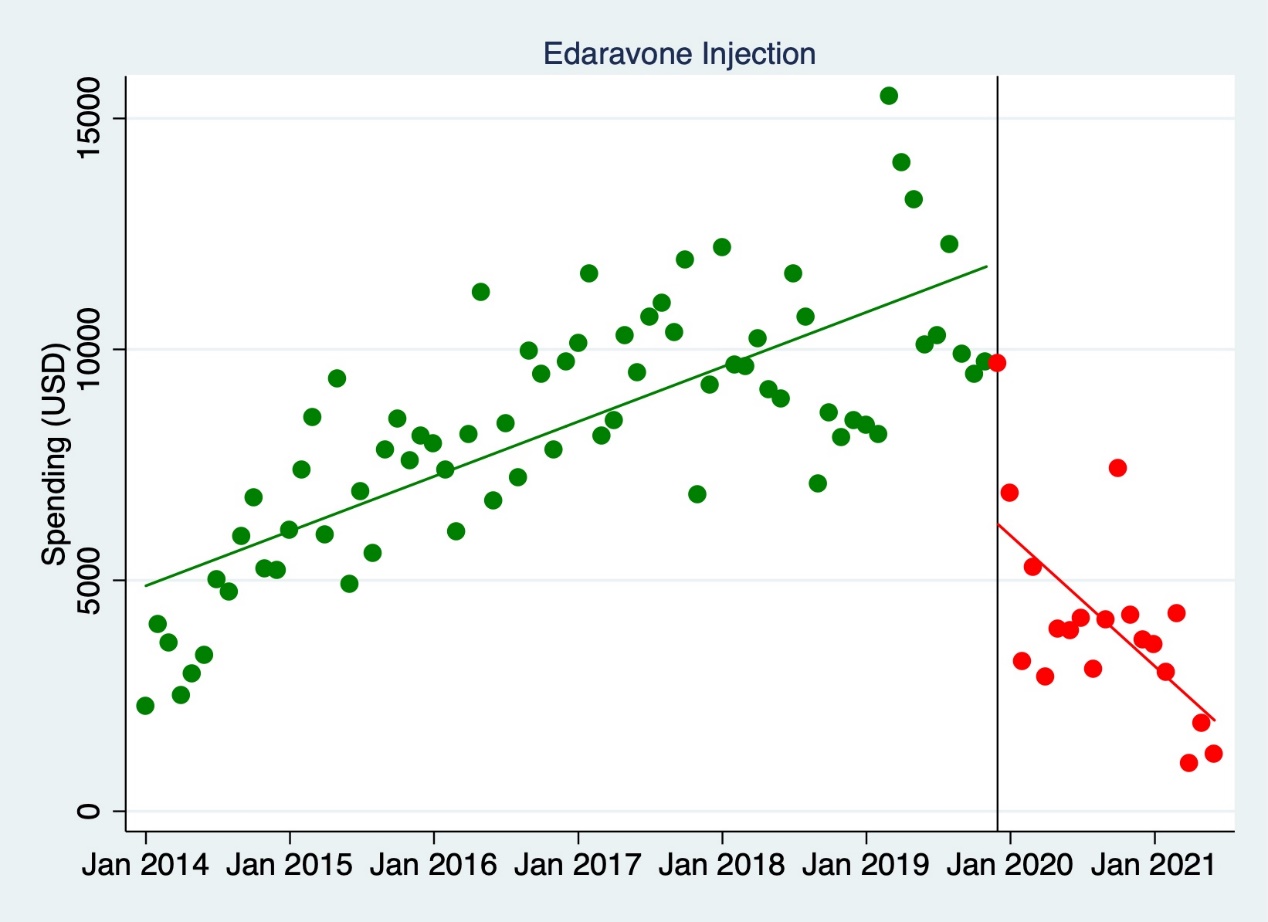


**(E)** Monthly spending of Edaravone Injection


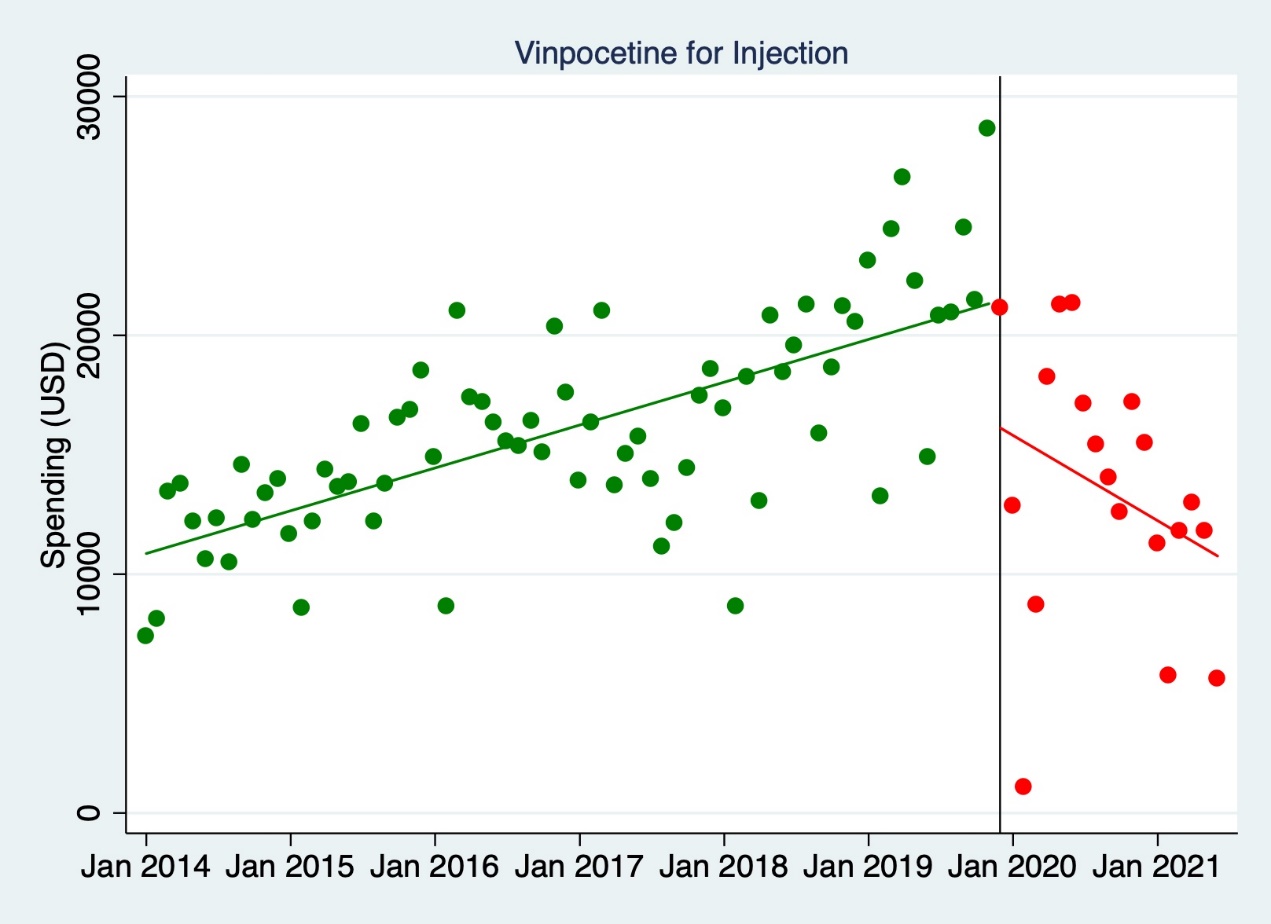


**(F)** Monthly spending of Vinpocetine for Injection


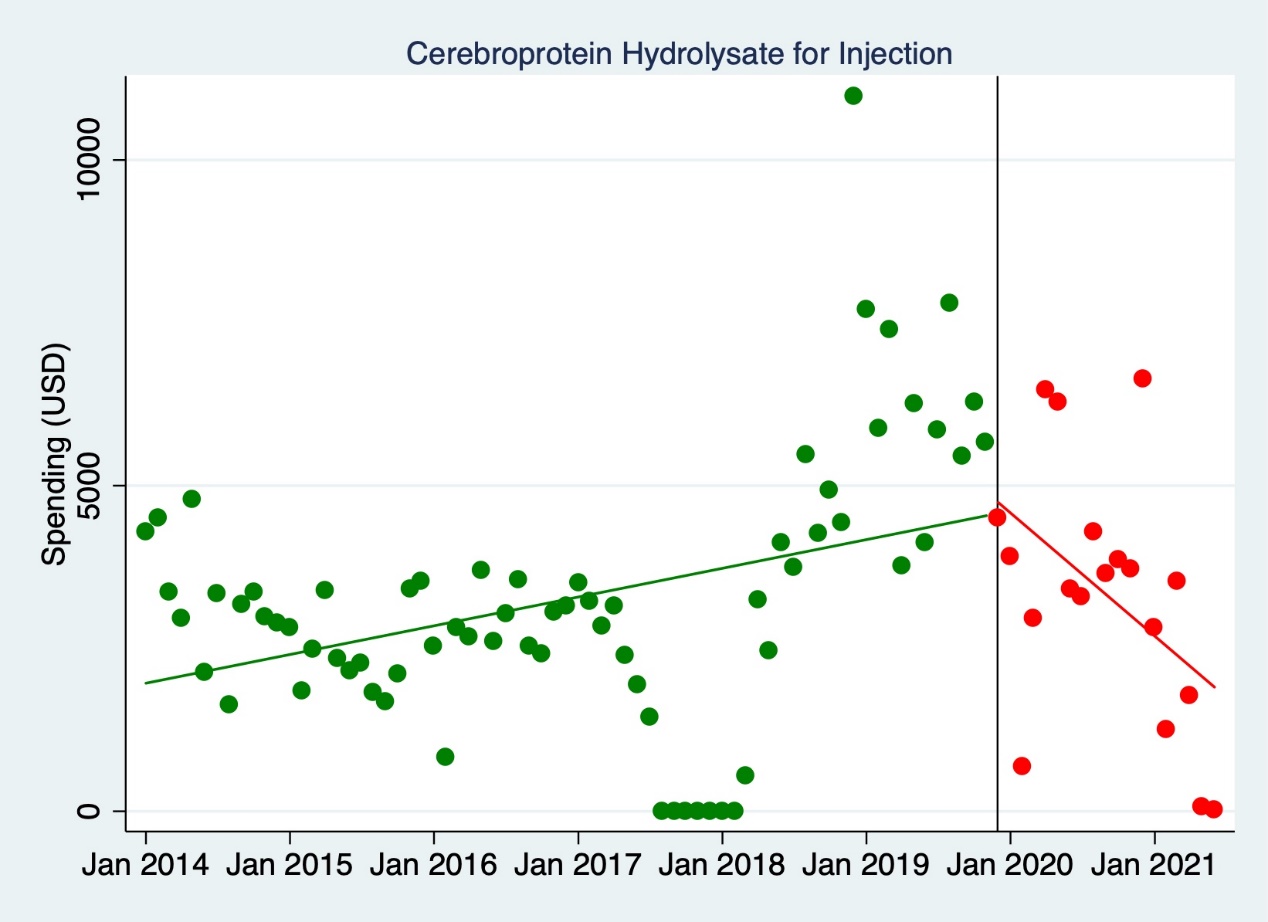


**(G)** Monthly spending of Cerebroprotein Hydrolysate for Injection


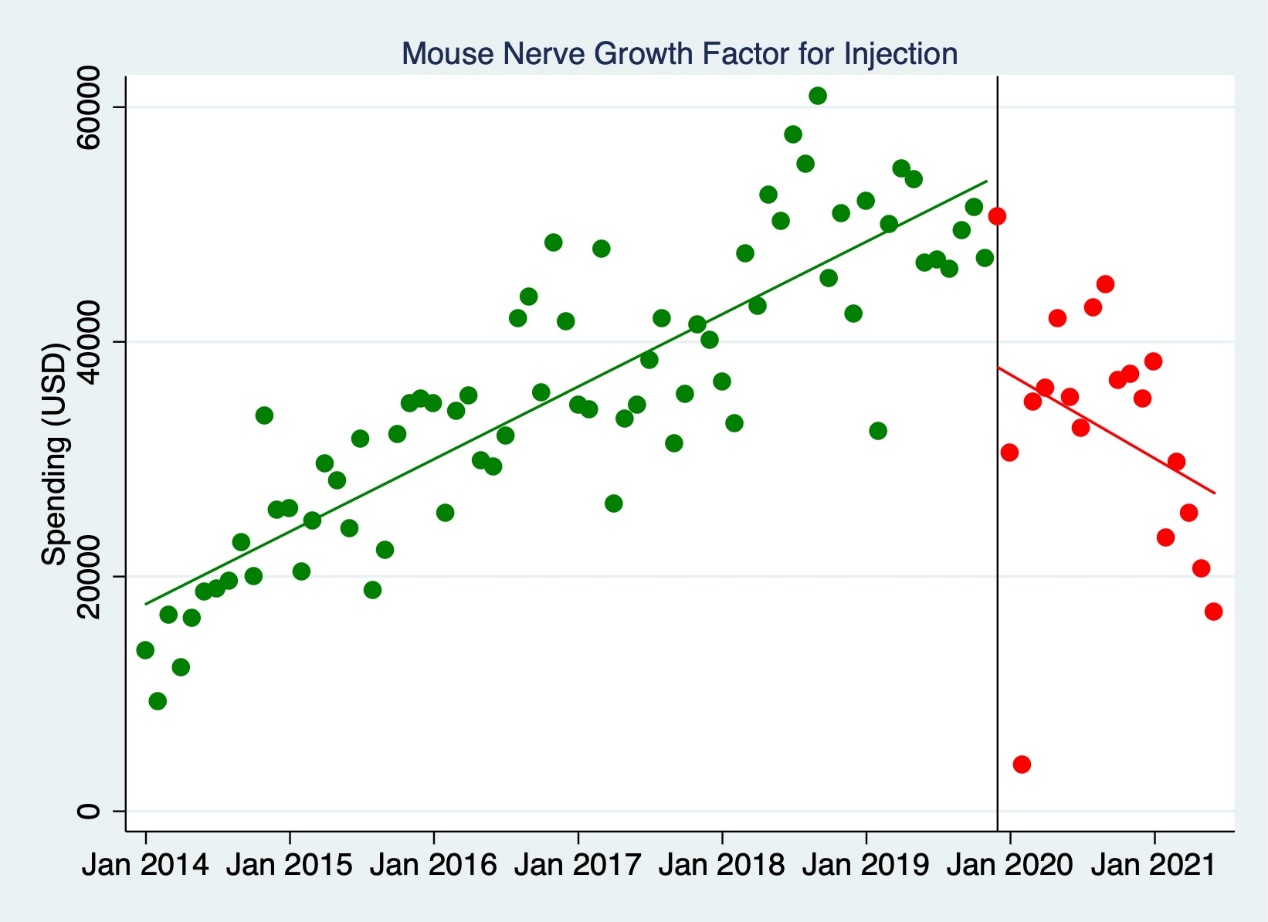


**(H)** Monthly spending of Mouse Nerve Growth Factor for Injection


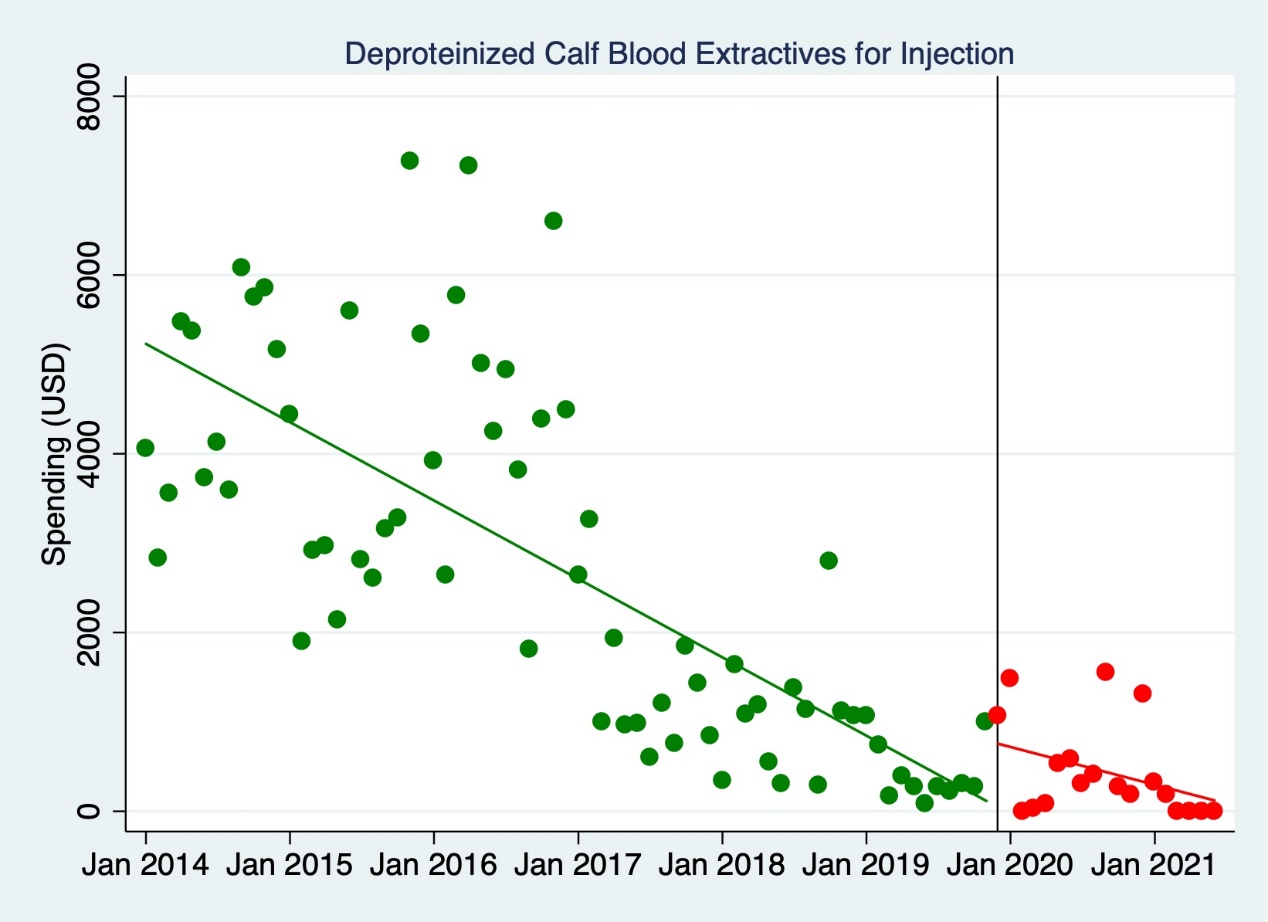


**(I)** Monthly spending of Deproteinized Calf Blood Extractives for Injection


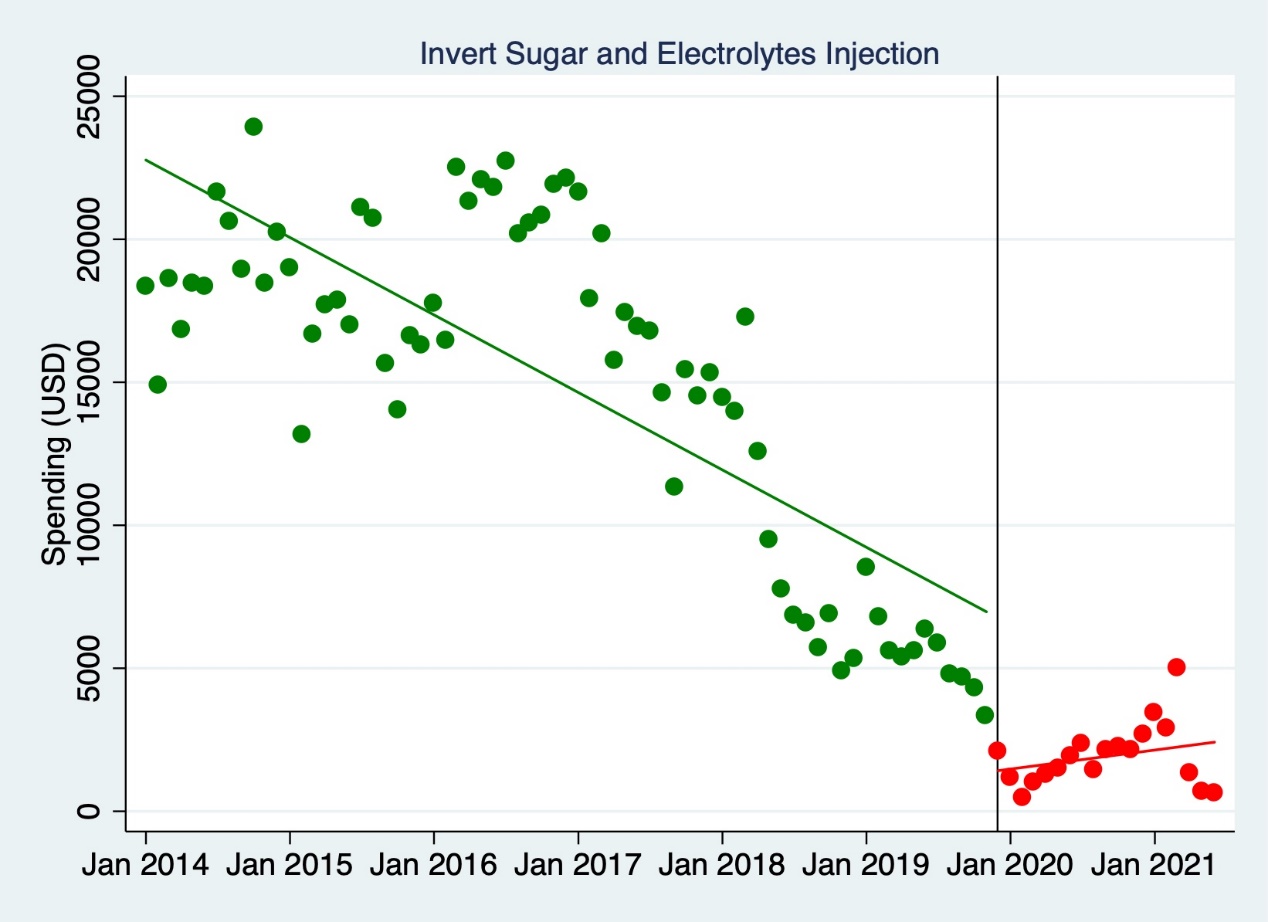


**(J)** Monthly spending of Invert Sugar and Electrolytes Injection

**FIGURE S2** Interrupted Time Series analyses for spending of ten national key monitoring drugs
