# Supplementary material for: The Impact of a National Stewardship Policy on the Usage Patterns of Key Monitoring Drugs in a Tertiary Teaching Hospital: An Interrupted Time Series Analysis
Source: Front Pharmacol. 2022 Feb 18;13:847353. doi: 10.3389/fphar.2022.847353 (PMC8895446; doi:10.3389/fphar.2022.847353)
Supplement: Supplementary file 3 [file Table2.docx]

**TABLE S2** Interrupted Time Series analyses for spending (USD) of ten national key monitoring drugs

| **Drug** | **Trend**  **Prior to Policy** | **95% CI** | **Dec 2019** | **95% CI** | **Trend  Dec 2019 –**  **Jun 2021** | **95% CI** | **Constant** | **95% CI** |
| --- | --- | --- | --- | --- | --- | --- | --- | --- |
| Salviae Miltiorrhizae and  Ligustrazine Hydrochloride Injection | 67.18 | 12.43 - 121.93 | -6,654.31 | -9283.95 - -4024.66 | -639.41 | -849.47 - -429.36 | 9,766.80 | 7007.75 - 12525.86 |
| Monosialotetrahexosylganglioside  Sodium Injection | 279.08 | 174.04 - 384.13 | -13,093.40 | -18401.8 - -7785 | -178.93 | -381.35 - 23.48 | 2,774.04 | -772.27 - 6320.36 |
| Alprostadil Injection | 222.61 | 143.8 - 301.43 | -16,514.57 | -23169.69 - -9859.44 | -588.85 | -1221.51 - 43.8 | 7,183.64 | 4244.19 - 10123.09 |
| Deproteinised Calf Blood  Serum Injection | 785.47 | 565.55 - 1005.4 | -30,186.17 | -50638.55 - -9733.78 | -1,511.08 | -3019.84 - -2.32 | 32,411.07 | 23335.23 - 41486.91 |
| Edaravone Injection | 98.76 | 66.65 - 130.87 | -5,686.44 | -8049.62 - -3323.26 | -334.15 | -463.17 - -205.13 | 4,780.04 | 3512.59 - 6047.48 |
| Vinpocetine for Injection | 149.52 | 115.41 - 183.64 | -5,376.42 | -11267.23 - 514.38 | -445.68 | -848.57 - -42.78 | 10,707.81 | 9634.88 - 11780.74 |
| Cerebroprotein Hydrolysate  for Injection | 36.78 | -5.33 - 78.89 | 162.32 | -2638.61 - 2963.25 | -194.18 | -357.47 - -30.89 | 1,927.96 | 721.81 - 3134.11 |
| Mouse Nerve Growth Factor  for Injection | 514.68 | 421.19 - 608.17 | -16,398.55 | -27625.44 - -5171.65 | -1,108.28 | -2027.02 - -189.55 | 17,132.80 | 13807.67 - 20457.92 |
| Deproteinized Calf Blood  Extractives for Injection | -73.08 | -89.56 - -56.6 | 712.62 | 36.9 - 1388.35 | 37.79 | 1.87 - 73.7 | 5,305.17 | 4371.31 - 6239.04 |
| Invert Sugar and Electrolytes  Injection | -225.70 | -304.5 - -146.89 | -5,328.73 | -8785.69 - -1871.77 | 280.87 | 149.17 - 412.58 | 23,001.02 | 19580 - 26422.04 |
