# Supplementary material for: The Impact of a National Stewardship Policy on the Usage Patterns of Key Monitoring Drugs in a Tertiary Teaching Hospital: An Interrupted Time Series Analysis
Source: Front Pharmacol. 2022 Feb 18;13:847353. doi: 10.3389/fphar.2022.847353 (PMC8895446; doi:10.3389/fphar.2022.847353)
Supplement: Supplementary file 4 [file DataSheet2.docx]

**Supplement 2**

**Interventions of NKMDs prescribing stewardship at Xi’an People’s Hospital (Xi’an Fourth Hospital)**

In this study, the catalogue of NKMDs was published and multidimensional interventions measures were formulated in November 2019 at the study hospital. Multidimensional interventions measures were implemented constantly by clinical pharmacists from December 2019 to June 2021. The specific interventions are described as below.

*1. Clarifying the responsibility of the management system*

A management leading group was set up by the medical department and the pharmaceutical department jointly, which was attached to the Pharmaceutical Management Professional Committee (PMPC) of the hospital. The management leading group was composed of administrators, clinicians, pharmacists and information staff. The leading group took responsibility of organizing the management of the whole process of the selection, procurement, prescription, dispensing, and clinical application of NKMDs. The catalogues of the NKMDs were published in the hospital. Regimens for the management and supervision of the rational use of NKMDs were also formulated. As a technical and functional section, the pharmacy department was responsible for training doctors on rational use of NMKDs, and for participation in consultations and monitoring (such as prescription review, data collection and report and feedback). Clinical pharmacists, who work in the clinical departments every day, gave their professional advice regarding NMKDs use directly to doctors. Information staff were responsible for technical implementation of the monitoring system. The medical department performed their respective duties for the NKMDs prescribing stewardship. Additionally, the director of the clinical department was identified as the person responsible for the rational use of NKMDs.

*2. Formulating regulations on the guidelines for clinical application of NMKDs*

The management group integrated all expert opinions and formulated regulations on the principles of clinical application of NMKDs. The regulations were determined based on the instructions provided by the manufacturer and evidence-based medical practice and issued by the PMPC. Thus, a doctor could determine whether the application of NKMDs was reasonable for patients according to the regulations, and the regulations were also the evaluation criteria on NMKDs use.

*3. Implementing educational programs*

All clinicians were convened to learn the Prescription Management Measures and the Guiding Principles for Clinical Application of NKMDs. The regulations required that physicians would not be given NMKDs prescribing privileges until after completing training on rational use of NMKDs. Clinical pharmacists were responsible for training clinicians on rational use of NMKDs in the lecture hall or clinical departments. The training was offered monthly, and all clinicians were required to attend the training at least once. Clinicians were required to prescribe NKMDs in a rational manner, including strict adherence to drug instructions aiming to not arbitrarily expand the indications of drug use, change the course or the dosage of treatment.

*4. Implementing prescription evaluation and audit, reward, and punishment system*

Clinical pharmacists would contact the doctors and provide face-to-face guidance in real-time to modify the prescriptions when unreasonable prescriptions were observed. Meanwhile, retrospective rationality evaluation of NMKDs prescriptions for patients was performed monthly by clinical pharmacists. Inappropriate prescriptions were flagged in the NMKDs Monitoring Report published by the pharmacy department each month. This report was made available to all medical staff and was provided to the prescriber through the medical department. After a monthly prescription audit, the management group updated the performance appraisal system for NMKDs use, which indicated the circumstances to be rewarded or penalized. Subsequently, drugs with persistent or prominent incidence of irrational use would be ranked, have usage rectified within a deadline, or would be removed from the supply catalogues of the hospital. According to the frequency and severity of inappropriate prescriptions, some doctors would be fined.

*5. Cooperation with information department*

Almost all prescriptions are initiated electronically, via a computer-based online stewardship strategy developed for clinicians. With the support of the information department, NKMDs were marked with an eye-catching ‘Supervision’ symbol in front of the drug names in hospital information system (HIS), indicating that this drug was monitored as NKMDs. With the training provided by clinical pharmacists, clinicians were reminded to pay more attention to the indications while prescribing. These could greatly influence and restrict the prescription of NKMDs. The monitoring system could be used to analyze and evaluate the clinical use of NMKDs, and regular monitoring reports and related early warning information of extraordinary varieties were issued.
