# Supplementary material for: The Impact of a National Stewardship Policy on the Usage Patterns of Key Monitoring Drugs in a Tertiary Teaching Hospital: An Interrupted Time Series Analysis
Source: Front Pharmacol. 2022 Feb 18;13:847353. doi: 10.3389/fphar.2022.847353 (PMC8895446; doi:10.3389/fphar.2022.847353)
Supplement: Supplementary file 5 [file DataSheet1.docx]

**Supplement 1**

**The instructions of 10 NMKDs in our study**


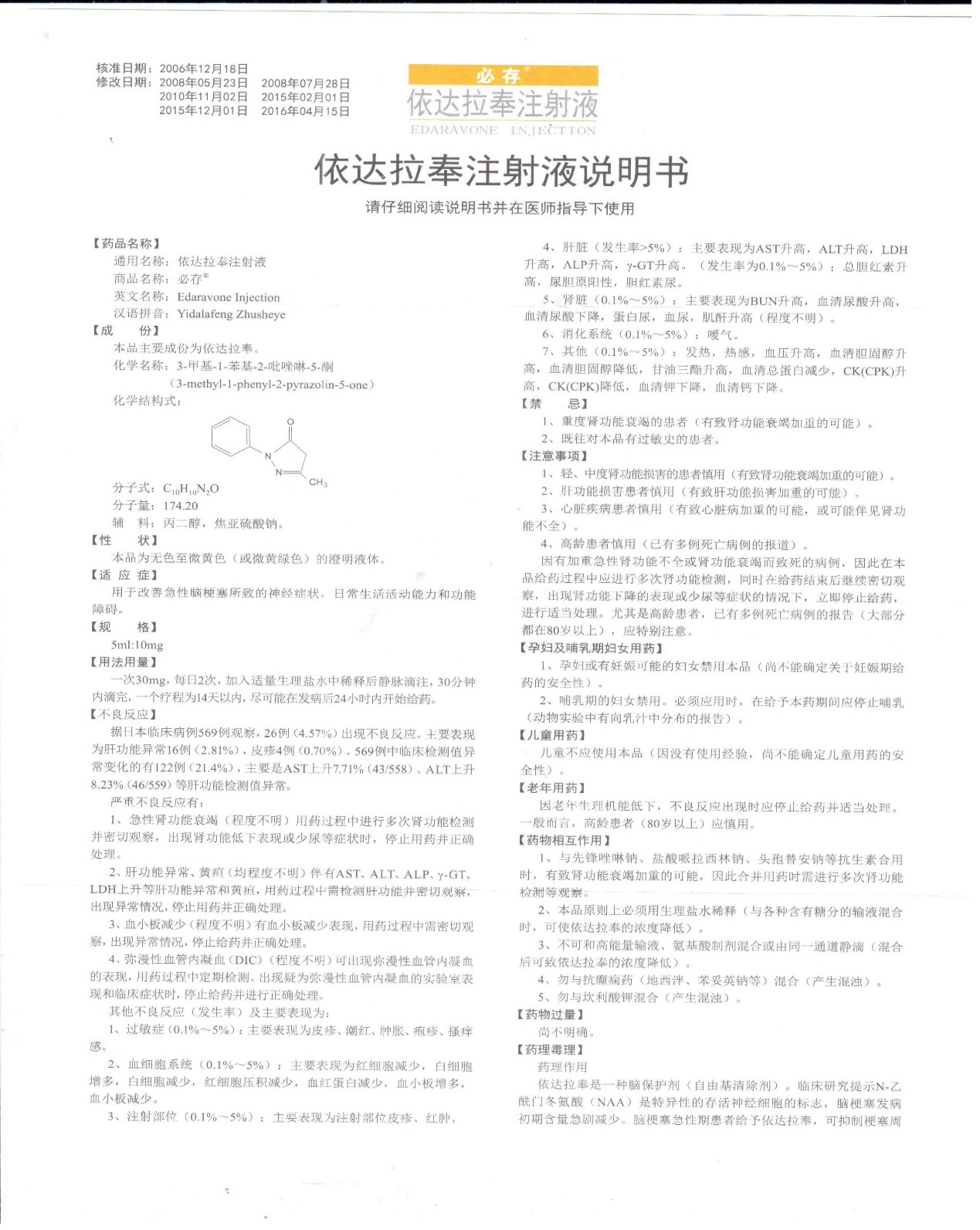


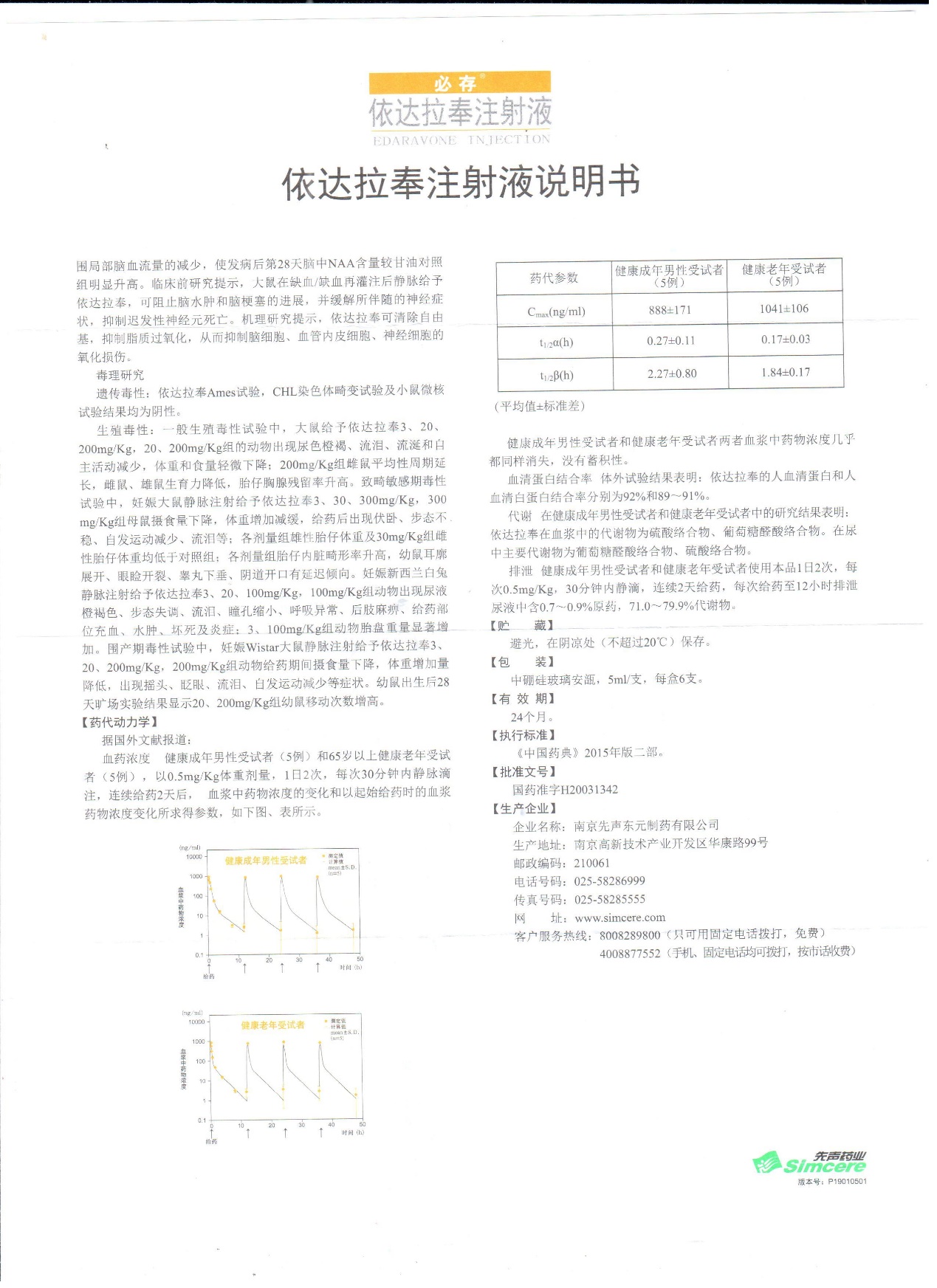


**(A)** The instruction of Edaravone Injection


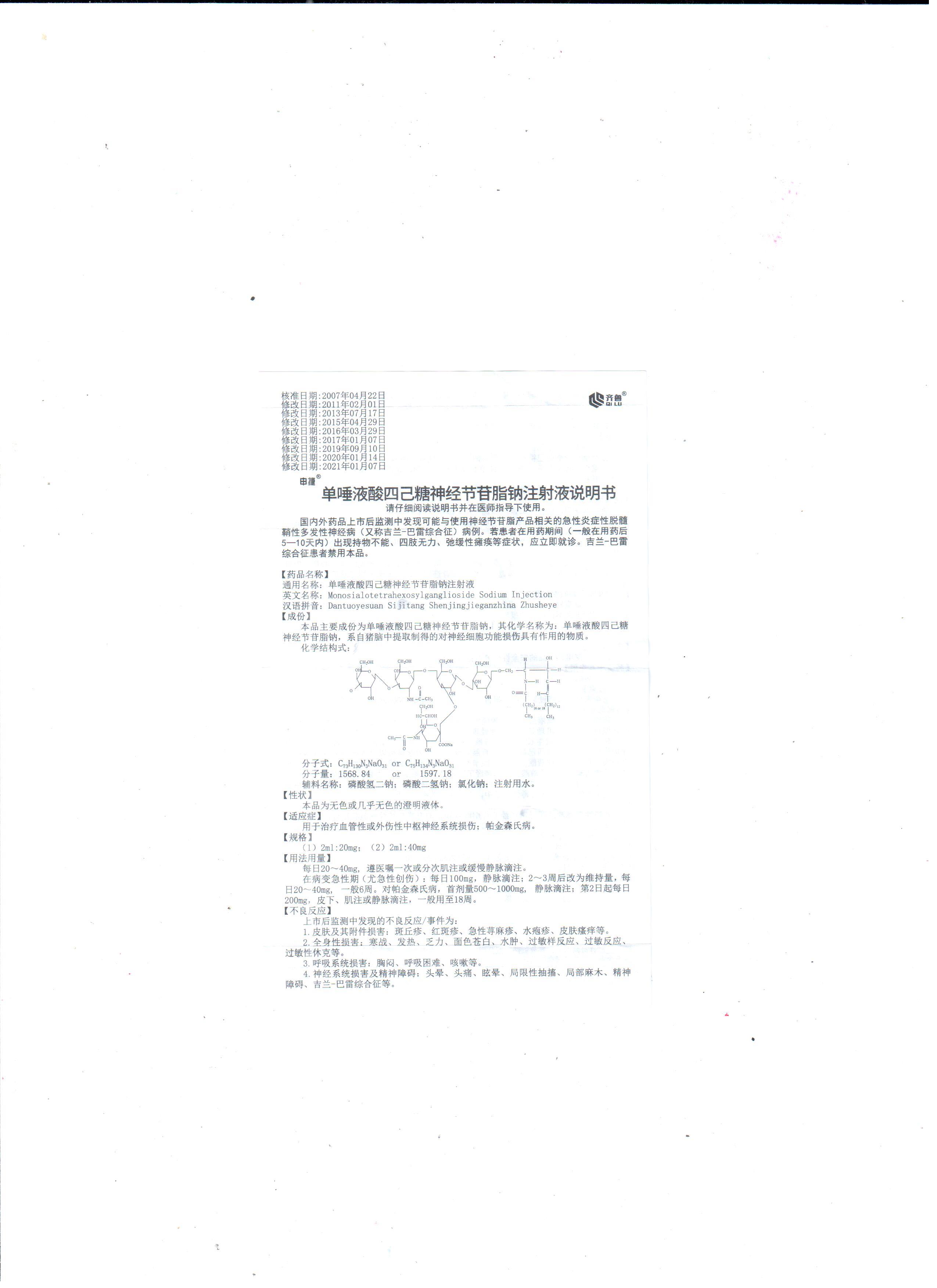


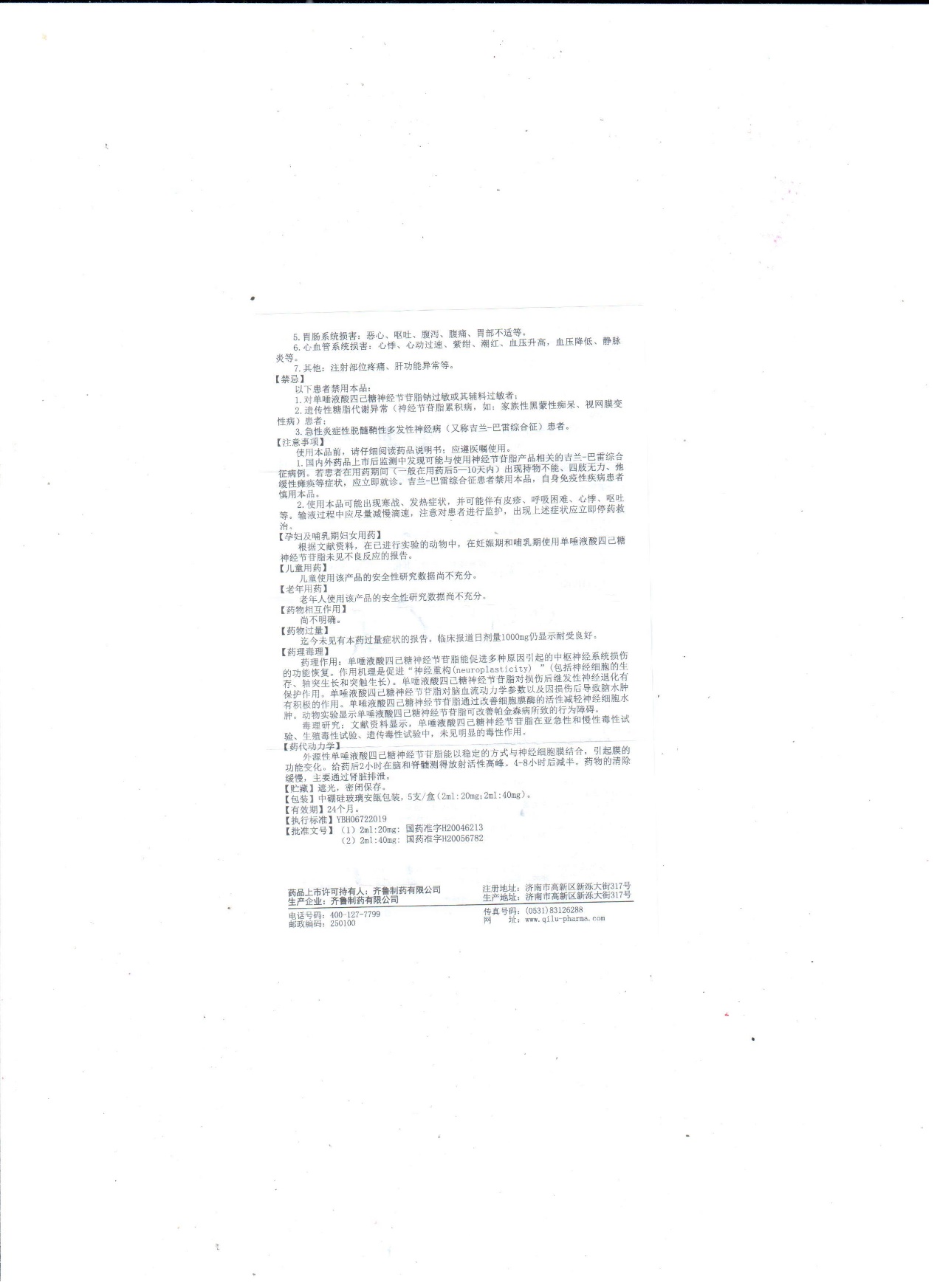


**(B)** The instruction of Monosialotetrahexosylganglioside Sodium Injection


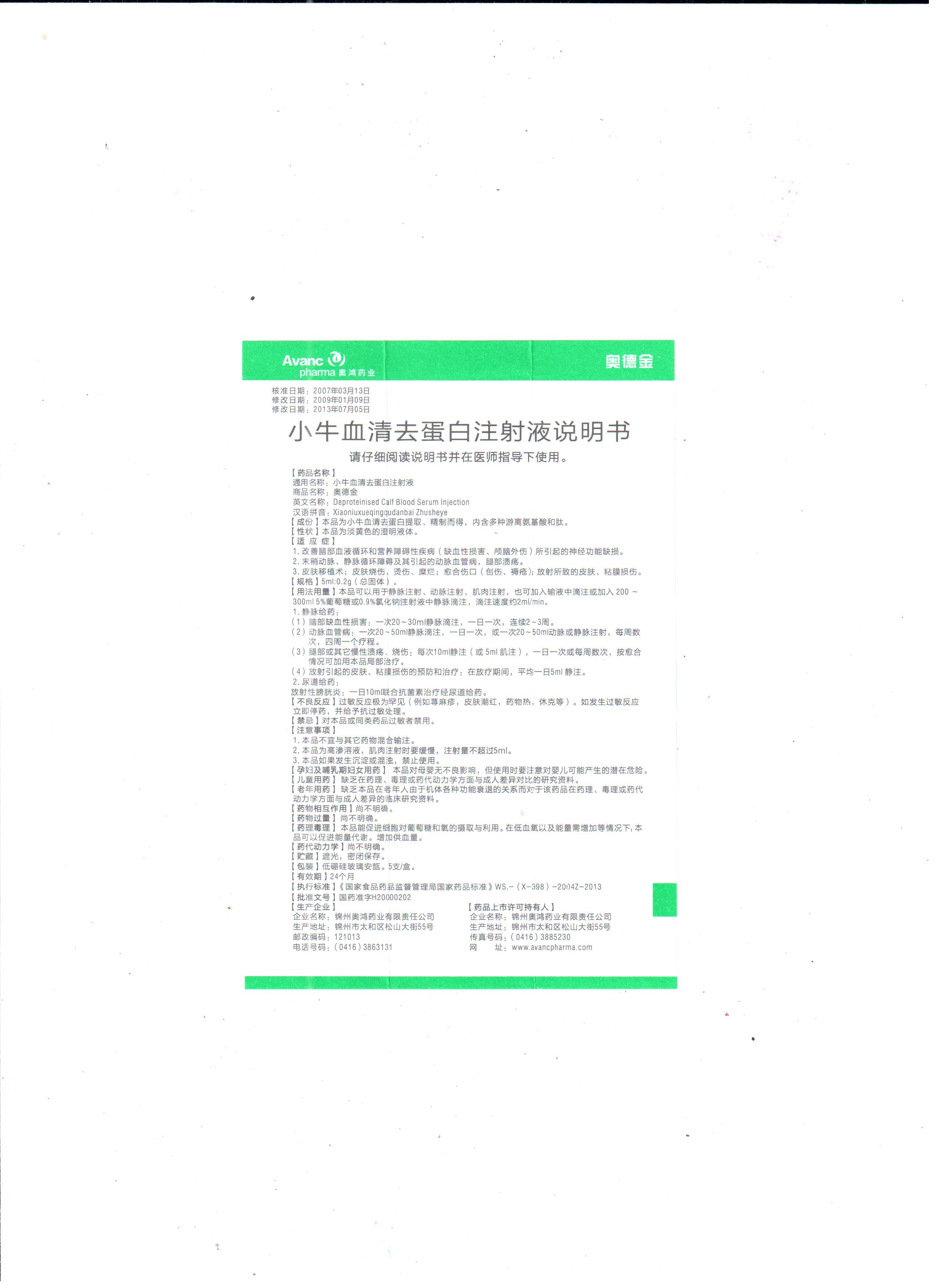


**(C)** The instruction of Deproteinised Calf Blood Serum Injection


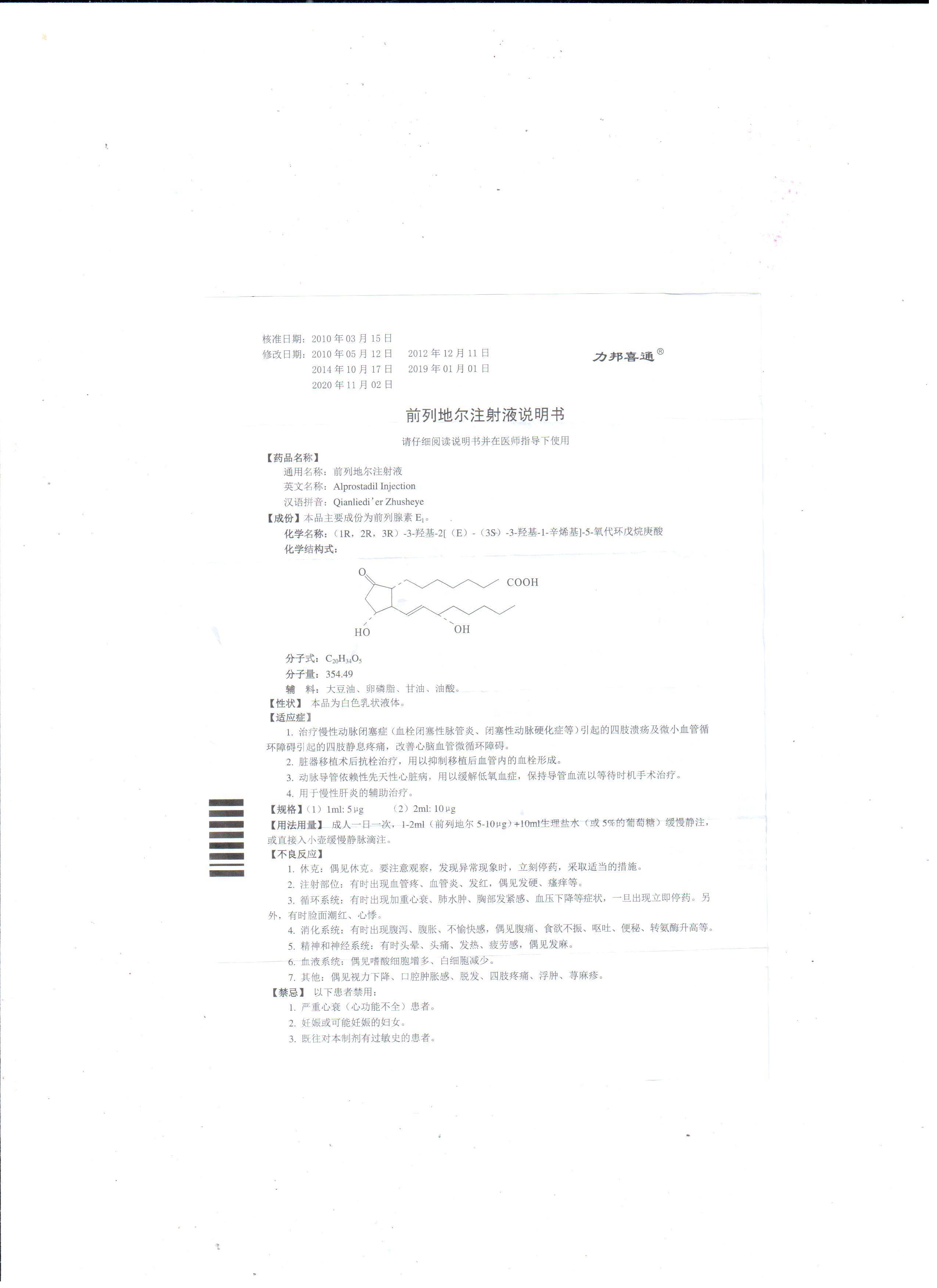


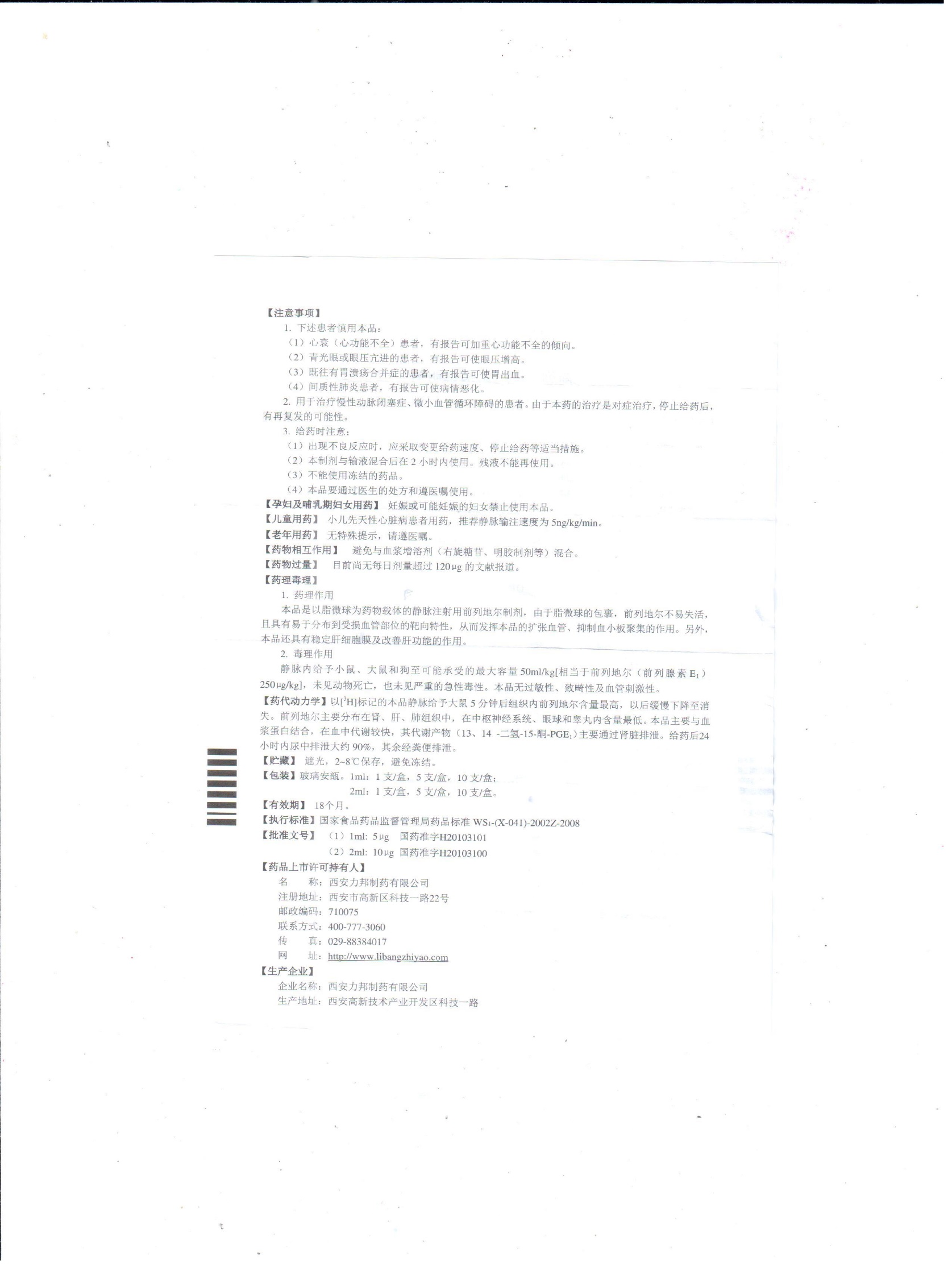


**(D)** The instruction of Alprostadil Injection


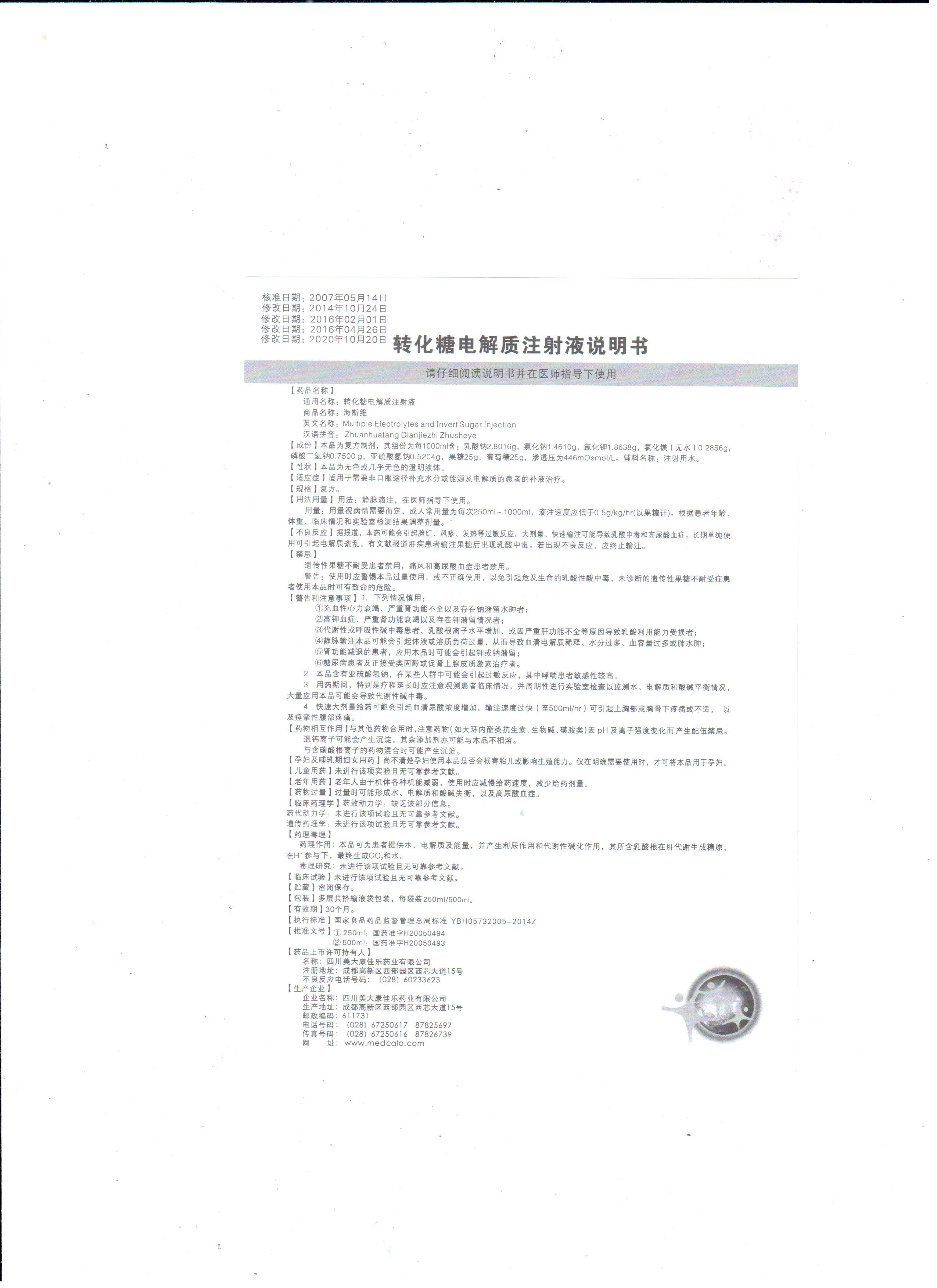


**(E)** The instruction of Invert Sugar and Electrolytes Injection


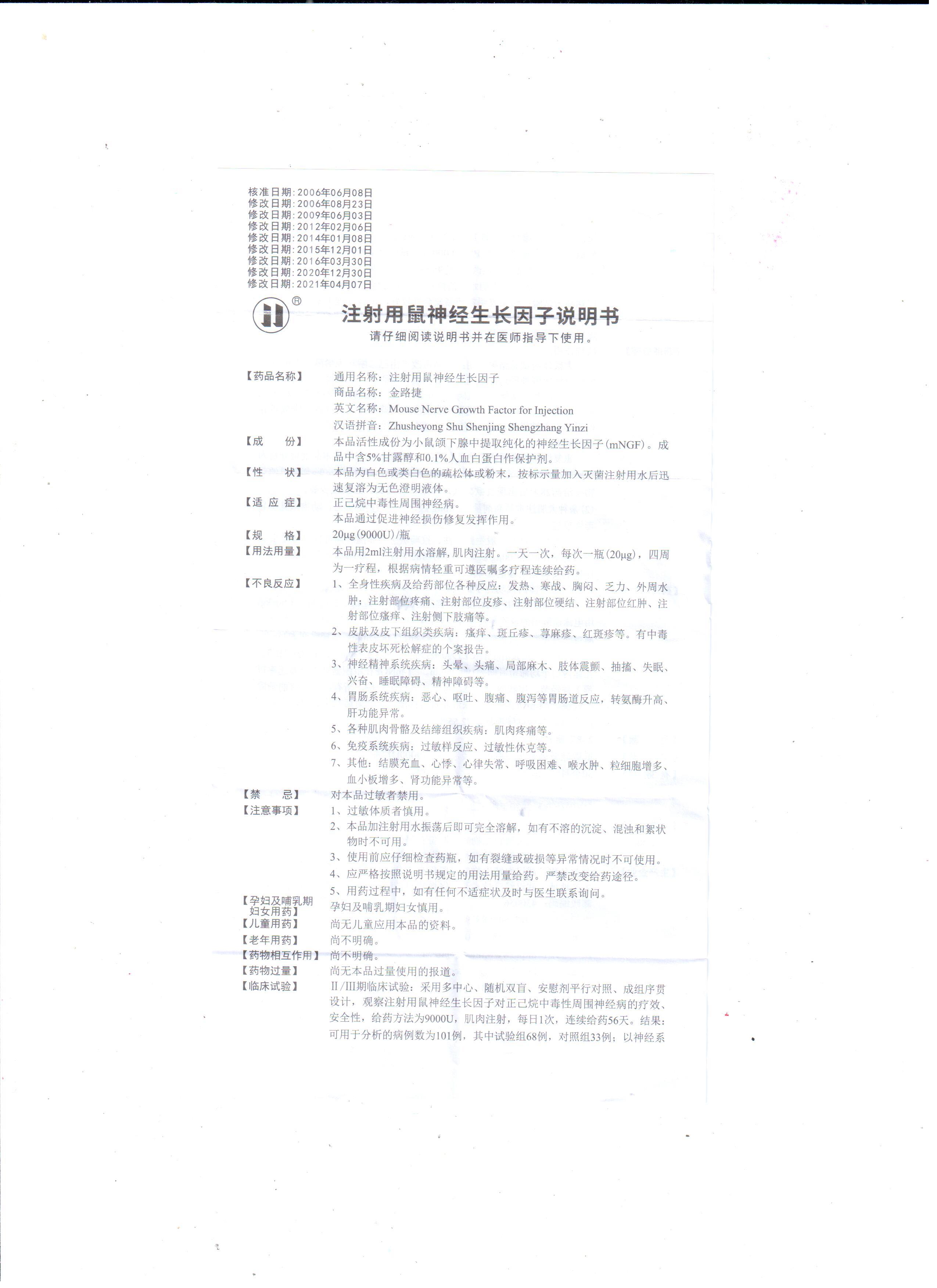


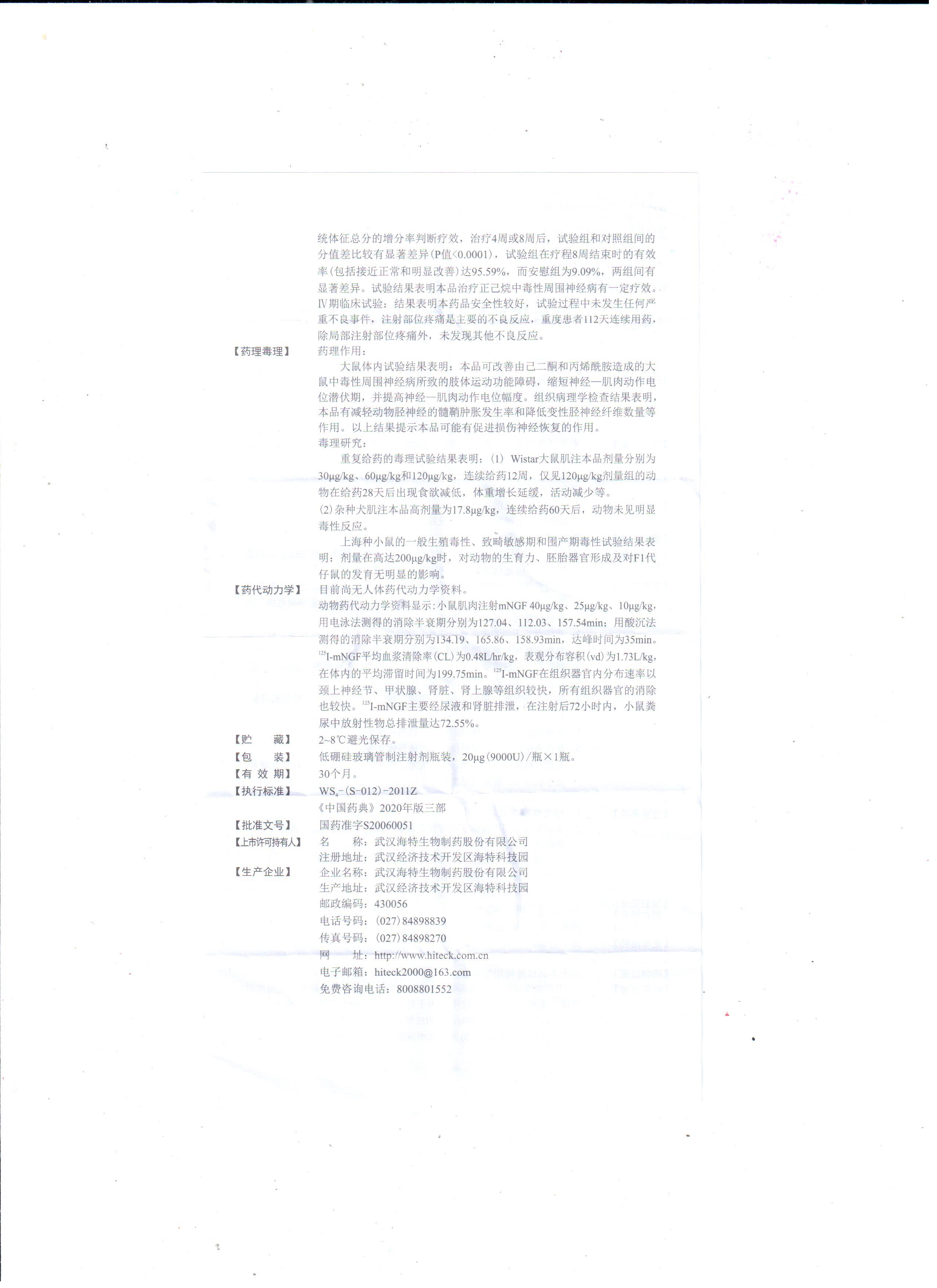


**(F)** The instruction of Mouse Nerve Growth Factor for Injection


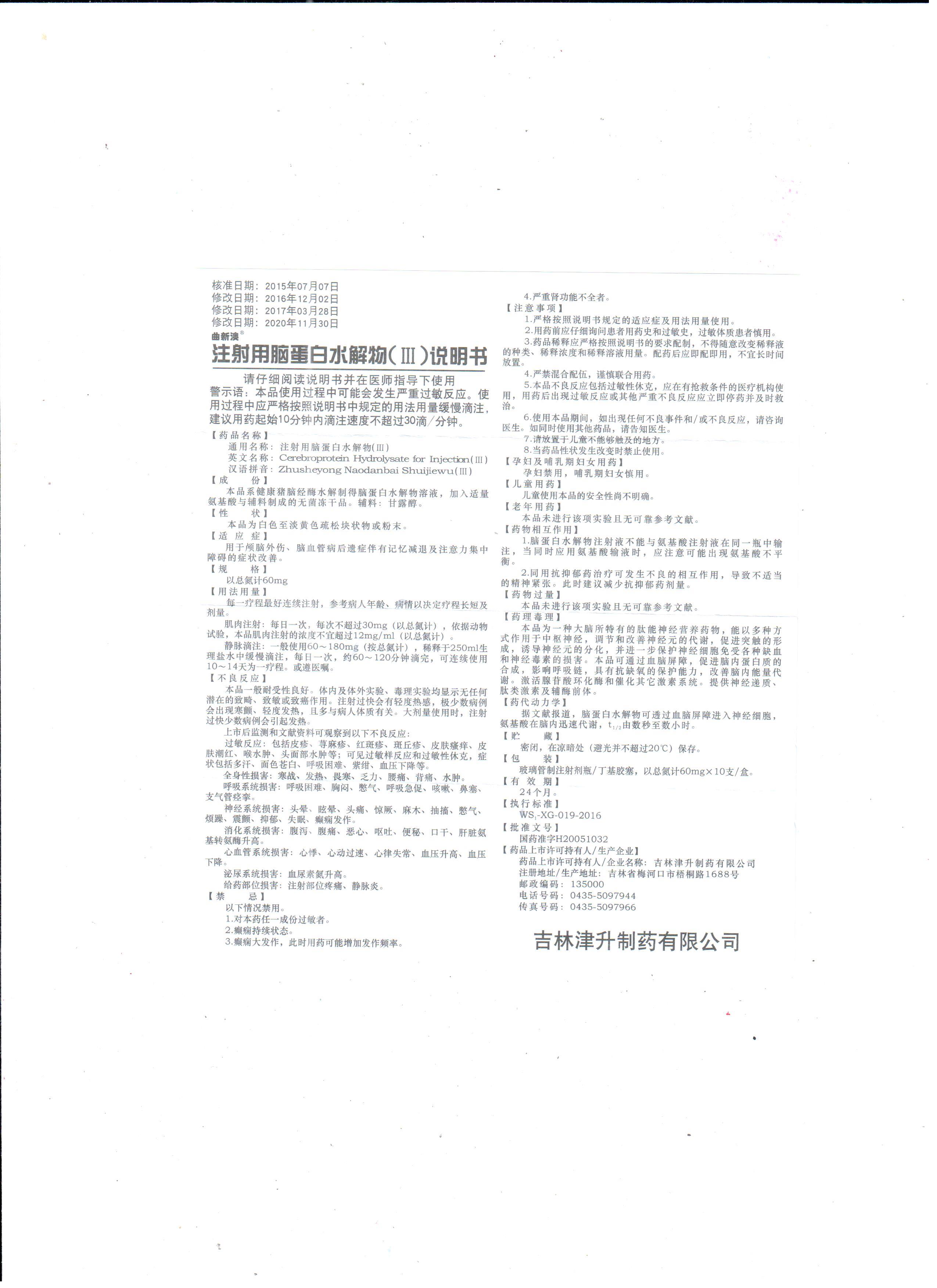


**(G)** The instruction of Cerebroprotein Hydrolysate for Injection


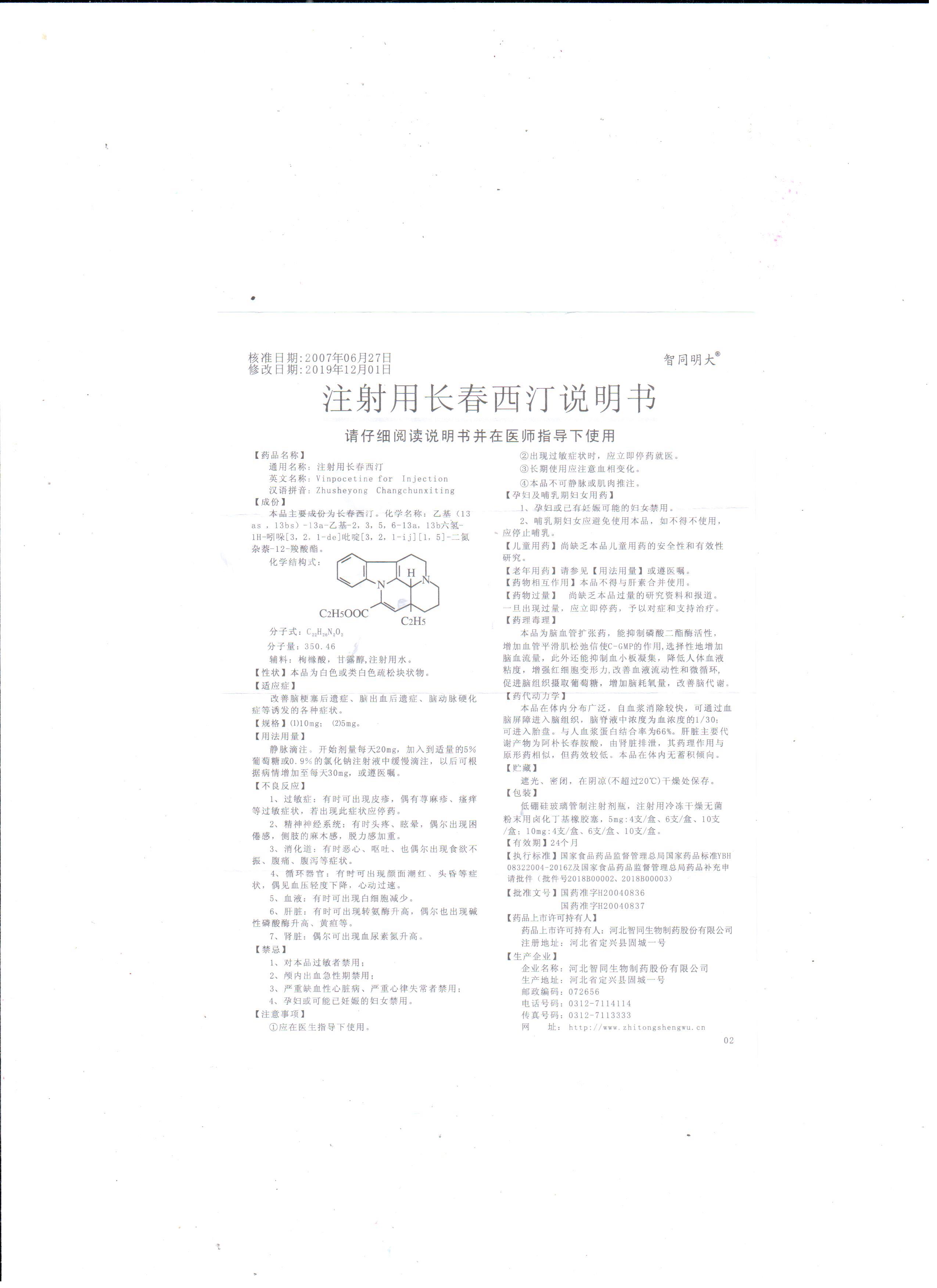


**(H)** The instruction of Vinpocetine for Injection


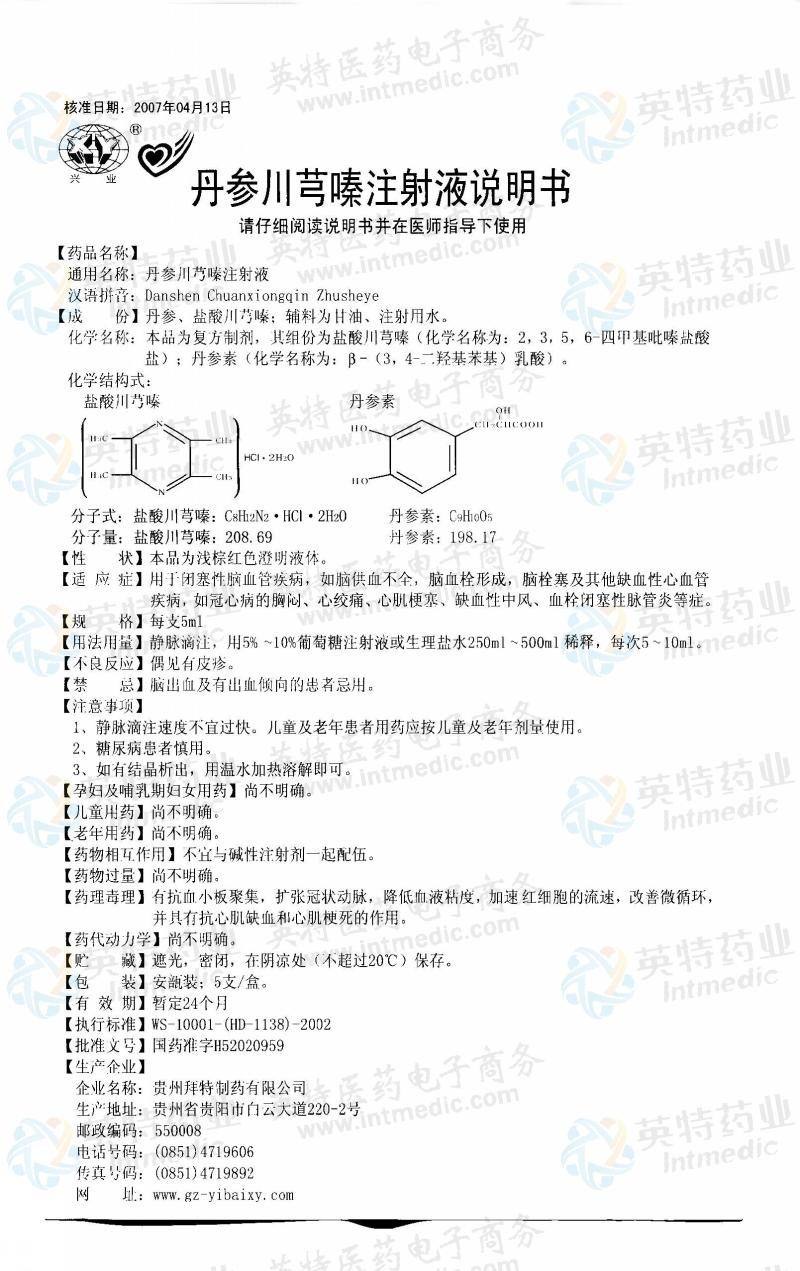


**(I)** The instruction of Salviae Miltiorrhizae and Ligustrazine Hydrochloride Injection


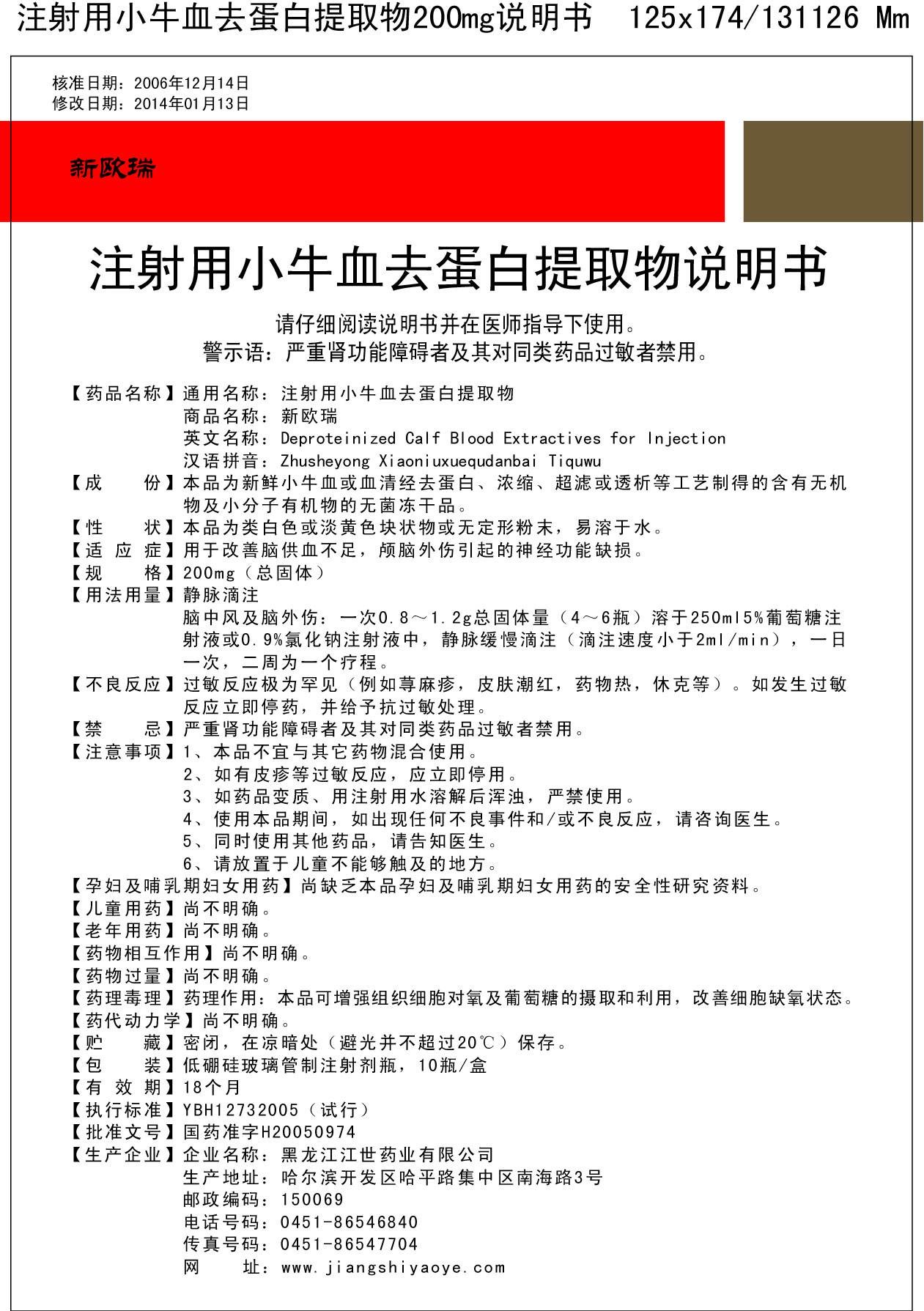


**(J)** The instruction of Deproteinized Calf Blood Extractives for Injection
